# Cancer systems immunology

**DOI:** 10.7554/eLife.53839

**Published:** 2020-07-13

**Authors:** Nathan E Reticker-Flynn, Edgar G Engleman

**Affiliations:** 1 Department of Pathology, Stanford University School of MedicineStanfordUnited States; 2 Division of Immunology and Rheumatology, Department of Medicine, Stanford University School of MedicineStanfordUnited States; 3 Stanford Cancer Institute, Stanford UniversityStanfordUnited States; PfizerUnited States; Keio University School of MedicineJapan

**Keywords:** systems biology, tumor immunology, cancer systems biology, systems immunology, tumor immunotherapy

## Abstract

Tumor immunology is undergoing a renaissance due to the recent profound clinical successes of tumor immunotherapy. These advances have coincided with an exponential growth in the development of –omics technologies. Armed with these technologies and their associated computational and modeling toolsets, systems biologists have turned their attention to tumor immunology in an effort to understand the precise nature and consequences of interactions between tumors and the immune system. Such interactions are inherently multivariate, spanning multiple time and size scales, cell types, and organ systems, rendering systems biology approaches particularly amenable to their interrogation. While in its infancy, the field of ‘Cancer Systems Immunology’ has already influenced our understanding of tumor immunology and immunotherapy. As the field matures, studies will move beyond descriptive characterizations toward functional investigations of the emergent behavior that govern tumor-immune responses. Thus, Cancer Systems Immunology holds incredible promise to advance our ability to fight this disease.

## Introduction

Systems Biology is an interdisciplinary field that aims to interrogate and predict complex behaviors of multivariate biological systems. It employs quantitative approaches to understand the integrated behaviors of multiple biological components. In contrast to reductionist approaches, which seek to identify how individual components affect particular phenotypes, systems biology attempts to query the simultaneous responses of many elements to uncover how they work in concert to elicit a given response. It is predicated upon the belief that many biological processes cannot be comprehensively understood by analyses of individual components alone (e.g. a single molecule, cell, etc.), but rather require a holistic appreciation of entire networks and systems (e.g. signaling networks, heterotypic cell-cell interactions, physiologic interplay between organs, etc.). By combining mathematical modeling and computation with experimental and clinical data, systems biologists can construct a framework for understanding the multiscale and temporal elements regulating biological responses and elucidate emergent behaviors.

While the discipline of systems biology became well established around 2000 (Ideker et al., 2001), its underlying concepts have been appreciated for over half a century (Waterman and Theory, 1968; Kitano, 2002). Indeed, some have suggested that the study of medicine, which requires an understanding of the complex interactions between multiple molecules, cell types, and organ systems in response to different treatments over time, represents an original implementation of Systems Biology (Germain, 2018). Nonetheless, recent advances in technologies and computational approaches have enabled researchers to query systems-level dynamics at scales not possible in previous decades (Hood et al., 2004).

Recently, researchers in the fields of both cancer biology and immunology have embraced systems approaches to advance their disciplines. In cancer biology, genomics and proteomics approaches have been implemented to identify the effects of defects in signaling networks on malignant transformation and progression (Sanchez-Vega et al., 2018; Mertins et al., 2016). Next-generation sequencing (NGS) has enabled studies of tumor heterogeneity and clonal evolution (Jacoby et al., 2015). In the United States, the National Cancer Institute formed the Cancer Systems Biology Consortium to promote applications of systems approaches to cancer.

Immunology represents a field that is readily amenable to systems level approaches. Deciphering the immune system requires an understanding of the interactions between numerous cell types, immune receptors, and cytokines as they traverse multiple anatomical locations and organ systems in order to orchestrate effective immune responses. While the multivariate components governing an immune response have been slowly elucidated through reductionist approaches, they have recently become subject to a much more comprehensive characterization through advances in modeling and high-throughput technologies (Davis et al., 2017).

Although the study of tumor immunology can be traced back at least to the advent of Coley’s toxins at the turn of the twentieth century (Starnes, 1992), the recent clinical successes of immunotherapies in the treatment of advanced stage cancers have catalyzed renewed interest in the field. Consequently, cancer systems immunology represents a new avenue of interrogation for understanding how the immune system interacts with tumors during tumorigenesis, progression, and treatment. Cancer systems biology and systems immunology have been reviewed elsewhere (Davis et al., 2017; Faratian, 2010; Suhail et al., 2019; Germain et al., 2011; Vera, 2015; Werner et al., 2014; Korsunsky et al., 2014; Kreeger and Lauffenburger, 2010; Chuang et al., 2010). In this review, we will discuss approaches to the nascent field of cancer systems immunology as well as their potential applications and current limitations.

## Applying systems biology to overcome challenges and discrepancies with animal models

Traditionally, animal models have served as critical tools to cancer biologists and immunologists as they try to decipher how tumors affect the host organism or how the immune response is orchestrated across multiple tissues, respectively. Nonetheless, animal models are frequently imperfect surrogates for human biology. While orthologous genes typically elicit similar functions across species, there are many instances where there exists a stark divergence in phenotypes for orthologs of different species (Gharib and Robinson-Rechavi, 2011; Koonin, 2005). Furthermore, there are even greater discrepancies between gene products that elicit the same functions, often reflecting a high degree of convergent evolution (Koonin, 2003). For example, inhibitory signaling in natural killer (NK) cells following recognition of major histocompatibility (MHC) class Ia molecules is achieved by Ly49 family members in mice but killer immunoglobulin-like receptors (KIRs) in humans (Lanier, 2005; Karlhofer et al., 1992; Moretta et al., 1990). In addition to differences in orthology, the cellular immune repertoires and the very existence of their associated effector molecules can vary significantly between species (Mestas and Hughes, 2004). All these factors frequently conspire to yield failed translation of therapeutic approaches when moving from preclinical models to human clinical trials (Denayer et al., 2014). As discussed throughout our report, systems biology offers potential solutions to this otherwise vexing problem through its ability to bridge data sets and models across species. Indeed, systems biology approaches have already provided predictive insights into human responses where preclinical models alone may be insufficient or inaccurate.

Computational biologists have developed a variety of tools to translate findings from preclinical models to humans when simple matching of orthologs is insufficient for predicting responses (Brubaker and Lauffenburger, 2020). To overcome differences in gene-to-function relationships and predict human responses from rodent data, researchers used Bayesian analysis of gene expression to define ‘functional orthologs’ across species (Chikina and Troyanskaya, 2011), and others have applied unsupervised and semi-supervised machine learning approaches to transcriptomic and proteomic data generated in rodent models to predict human responses (Brubaker et al., 2019). On collaborative initiatiative, termed SBV-IMPROVER (Systems Biology Verification for Industrial Methodology for PROcess VErification in Research), sought to develop computational methods capable of cross-species translation using multimodal datasets including transcriptomics, phosphoproteomics, and cytokine data (Poussin et al., 2014; Rhrissorrakrai et al., 2015). Solutions ranged from approaches using support vector machines, neural networks, random forest trees, and more, with no one algorithm outperforming the others across all datasets. These types of cross-species comparisons have been extended to single-cell analyses, wherein cell types can be defined separately in the different organisms and matched manually, through correlation analyses, or through the use of random forest machine learning (Tosches et al., 2018; Butler et al., 2018; Shafer, 2019; Elyada et al., 2019). Nonetheless, the choice of preclinical model is critical as a given model may not be as informative as another (Olson et al., 2018). For example, transplantable pancreatic cancer models did not predict the limited efficacy of gemcitabine as accurately as autochthonous models due to a lack of desmoplastic stroma in the transplantable setting (Olive et al., 2009).

Even with improved animal models, however, there are many instances where they fail to predict clinical responses, and only 8% of drugs entering clinical trials succeed in Phase I (Mak et al., 2014). Thus, the use of systems approaches, informed by existing data sets, to accurately predict human responses in the absence of accurate animal models represents an important opportunity to improve translation. Mathematical models have proven effective at accurately predicting many aspects of cancer biology ranging from growth kinetics and tumor evolution to responses to therapy (Altrock et al., 2015). For example, such models have tracked the development of resistance in CML (Michor et al., 2005) or have served as the basis for clinical trials altering dosing strategies (Norton and Simon, 1977; Citron et al., 2003). Critically, mathematical models have been used to inform clinical decisions. In an approach they term ‘adaptive therapy’, one group used an evolutionary game theory model to predict patient-specific treatment responses in patients with castration-resistant prostate cancer, modifying their treatment accordingly, and in doing so, extended the time to progression in these patients (Zhang et al., 2017a). This study highlights the profound impact systems-level mathematical modeling can impart on clinical decision-making. Systems immunology has been applied particularly in the field of vaccinology to reduce the reliance upon animal models (Davis et al., 2017), and these types of modeling approaches have recently been extended to cancer immunology to understand how treatment regimens can be tailored to improve immune responses (Park et al., 2019). By extending these approaches further, cancer systems immunology holds the potential to inform clinical approaches when animal models are insufficient.

## Technologies

**Figure 1.**
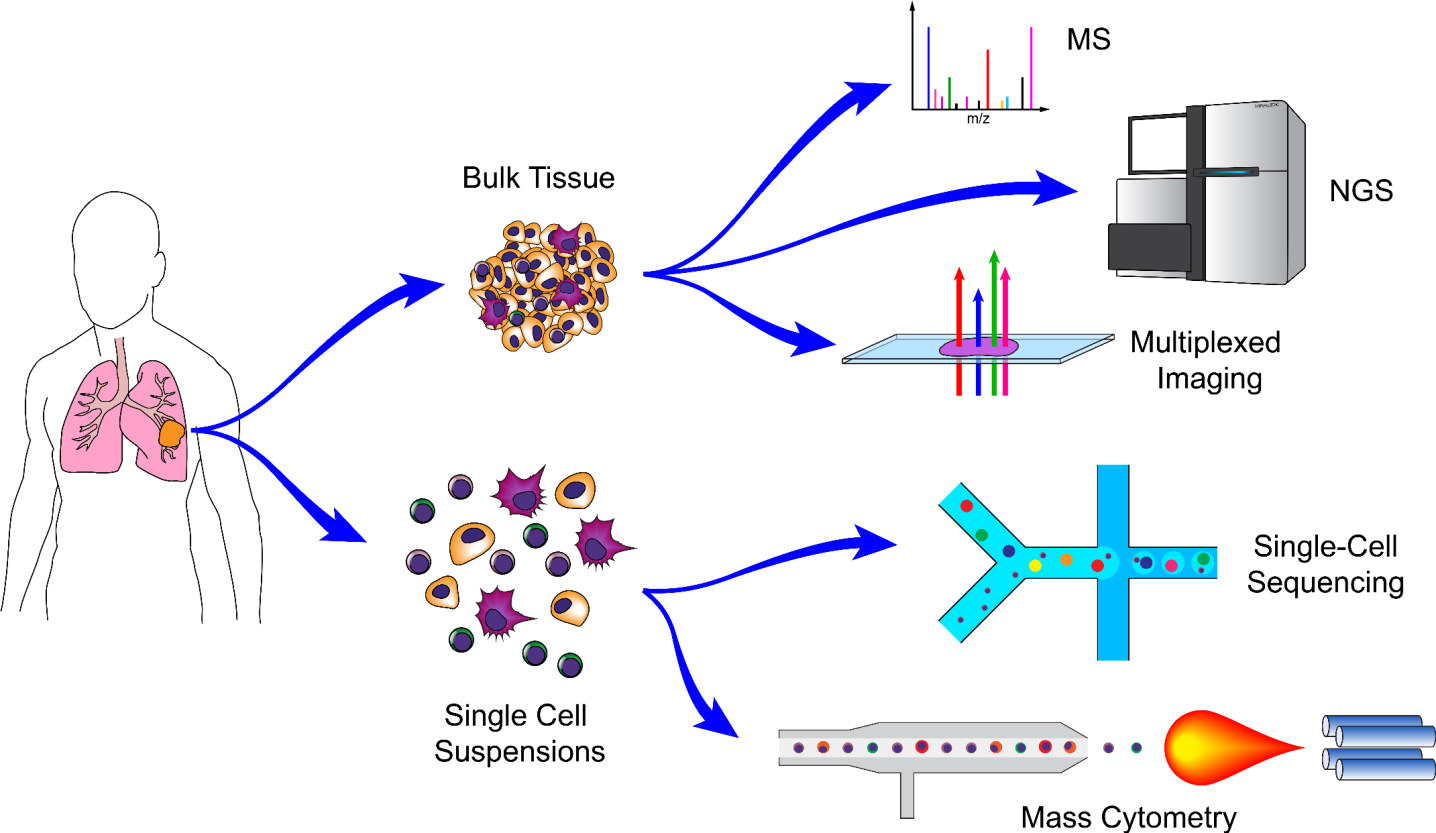
Technologies for cancer systems immunology. Technologies used in cancer systems immunology operate either on bulk tissue samples or single-cell suspensions. Conventional MS, NGS, and imaging platforms do not require tissue dissociation (although histology provides single-cell resolution). Droplet-based microfluidics and mass cytometry, in contrast, require cell suspensions, but generate high-dimensional data for individual cells.

The multivariate nature of systems biology has rendered it particularly applicable to comprehensive datasets derived from quantification of systems-level parameters (e.g. genomics, transcriptomics, metabolomics, proteomics, etc.). Indeed, the beginning of modern-era systems biology largely coincides with the Human Genome Project (Lander et al., 2001; Venter et al., 2001; International Human Genome Sequencing Consortium, 2004), which enabled researchers to interrogate the genome-wide contributions of mutations to diseases. It is worth noting that systems biology does not require the use of any of these advanced technologies, and many mathematical and computational models were derived from experimental evidence collected with conventional assays. Similarly, the use of these next-generation technologies for a particular study does not, in and of itself, constitute a systems biology approach. Frequently, such technologies are used to screen for targets that are subsequently subjected to conventional reductionist analyses (e.g. differential gene expression (DGE) analyses of RNA-seq data to identify a gene of interest). Such approaches alone do not provide a systems-level understanding of a particular phenomenon, as they do not describe emergent behavior that could not be uncovered with reductionist approaches. Nonetheless, a wide range of new technologies has enabled researchers to examine the breath and dynamics of entire systems in order to better understand the interplay of multiple elements and networks (Figure 1; Hood et al., 2004; Ideker, 2001). Here, we describe some of the major technologies adopted by cancer systems immunologists to uncover new biology. Strengths and weaknesses for the various genomic and epigenomic profiling technologies are highlighted in Tables 1–3.

**Table 1.** Genomic and transcriptomic profiling technologies.

Measurement	DNA	DNA	DRNA/RNA	RNA	RNA
Technology	WGS	WES	Amplicon (e.g. TCR, BCR, specific loci)	RNA-seq	Microarray
Strengths	• Captures coding and non-coding regions • may be more accurate in some exons as well • better coverage in low-complexity regions • no PCR step required	• Reduced cost of sequencing since restricted to 2% of genome	• Lower cost • greater sequencing depth	• Not limited to known genes with probes • can identify splice variants • can include ncRNA • can identify sequence variations (e.g. mutations)	• Can theoretically detect very low abundance transcripts at no additional cost
Weaknesses	• High cost	• Does not capture non-coding regions • may fail to capture some coding regions depending on probe hybridization • GC bias can be introduced due to PCR • hybridization bias can occur in regions with heterozygous SNVs	• Limited to specific regions (not genome-wide)	• Sequencing depth can limit the ability to detect low-abundance transcripts	• Probe bias • inability to compare relative abundance across genes • limited to known transcripts (for which there are probes)
Single-cell Version?	Y	Y	Y	Y (see Table 3)	Y* (very uncommon, Esumi et al., 2008)

**Table 2.** Epigenetic profiling technologies.

Measurement	Technology	Strengths	Weaknesses	Single-cell version?
Methylation	WGBS	• No a priori sequence selection	• High cost and may require higher coverage • cannot distinguish type of modification at cytosines	Y
Methylation	RRBS	• Lower cost	• Limited mainly to CpG islands • cannot distinguish type of modification at cytosines	Y
Protein Localization	ChIP-seq	• Genome-wide profiling of histone modifications and DNA-protein interactions (Histone H3 acetylation/methylation, TF binding site identification, SE identification)	• Survey only one type of interaction (protein) at once • lots of sources of noise/bias • requires good antibodies • requires input DNA and isotype controls • requires large input of cells	Y
Protein Localization	CUT&RUN	• Fewer input cells required than ChIP • less noise • fewer sequencing reads required • no cross-linking required	• Requires good antibody • potential for overdigesting DNA	Y (CUT&Tag, uliCUT&RUN)
Chromatin Accessibility	DNAse-seq	• Identify a range of *cis* and *trans* regulatory elements including TF binding sites	• High input cells requirement • more time-consuming that ATAC • sequence bias	Y
Chromatin Accessibility	ATAC-seq	• Identify a range of *cis* and *trans* regulatory elements including TF binding sites • minimal input cells required • increased sensitivity over DNAse-seq • simple protocol	• Footprint profiles can be less well-defined than DNAse-seq • potential mitochondrial DNA contamination	Y
Chromatin Accessibility	MNAse-seq	• Nucleosome occupancy and positioning • can be used to predict higher-order structure (e.g. 3D)	• Requires crosslinking • highly dependent on enzyme concentration • some sequence bias	Y
Chromatin Accessibility	FAIRE-seq	• No sequence bias • simple protocol • no enzymes required	• Requires crosslinking • lower resolution (crosslinking binds chromatin but also TFs) • large input cell requirement	N
3D Conformation	3C (Chromosome Conformation Capture)	• Identify single chromosomal interaction (one vs. one)	• limited resolution (by 6bp cutters) • laborious • PCR biases • high library complexity • single viewpoint	N
3D Conformation	4C (Circular 3C)	• Improved resolution over 3C • can identify very long range interactions • can identify all contacts for a locus (one vs. all)	• Biases from circularization • PCR biases • high input cell requirements • single viewpoint	N
3D Conformation	5C (3C Carbon Copy)	• Can identify many contacts for multiple loci (many vs. many)	• Bias introduced by probe ligation efficiencies • not all fragments can bind probes • all vs. all prohibitively expensive	N
3D Conformation	NG Capture-C	• Analyze hundreds of viewpoints • can identify PCR duplicates (low bias) • highest sensitivity and resolution • fewer input cells required	• Occasional non-specific interactions	N
3D Conformation	Hi-C	• Maps contacts across whole genome (all vs. all) • kilobase resolution	• Fewer contacts per fragment than 4C or Capture-C • higher resolution versions require extremely high sequencing depths	Y
3D Conformation and Protein Localization	ChIA-PET	• Combines 3D interactions with protein interactions	• Interactions defined by few reads • high input requirements • bias toward interactions with targeted protein	Y* (ChIA-Drop: single molecule, Zheng et al., 2019)
3D Conformation and Protein Localization	Hi-ChIP (and PLAC-seq)	• Lower input required • higher yield than ChIA-PET • higher signal to noise over Hi-C	• Bias toward interactions with targeted protein	N

**Table 3.** scRNA-seq Technologies.

Strengths and weaknesses of the ever-evolving compendium of scRNA-seq technologies and analysis packages have been evaluated reviewed extensively in Ziegenhain et al., 2017; Chen et al., 2019a; Haque et al., 2017. Here, we provide a basic overview of the strengths of the general approaches.			
Technology	Plate-based (e.g. Smart-seq2, MARS-seq)	Microfluidic capture (e.g. C1, Seq-well, CEL-seq2/C1)	Droplet (e.g. 10X, Drop-Seq)
Strengths	• Highest sensitivity (number of genes detected) • fewer multiplets • full-length transcripts possible	• High sensitivity (number of genes detected) • fewer multiplets • no sorting required	• Inexpensive (per cell) • profile high numbers of cells • can identify less frequent cell types • no sorting required • Can use UMIs
Weaknesses	• Requires sorting • low throughput • high cost per cell • not strand specific	• 3' Only • limited cell numbers • (typically) not strand-specific	• 3' Only • fewer genes/UMIs • more dropout

### Bulk sequencing technologies

The promise of the Human Genome Project and whole genome sequencing (WGS) has inspired the development of –omics technologies capable of characterizing the entirety of a particular attribute within a sample. Such approaches include, but are not limited to, genomics, transcriptomics, epigenomics, proteomics, metabolomics, lipidomics (Yang and Han, 2016), and glycomics (Bennun et al., 2016; Cummings and Pierce, 2014; Bertozzi and Sasisekharan, 2009), and their current manifestations typically utilize variations of next-generation sequencing (NGS) or mass spectrometry (MS). The bulk implementations of these technologies (i.e. those that require multiple cells as inputs – frequently, thousands to millions) were the precursors of many of the single-cell versions that have recently gained popularity. While these technologies on their own do not provide single-cell resolution, they frequently provide a degree of sensitivity that cannot yet be achieved at the single-cell level (e.g. most glycomics). Furthermore, the cost of these approaches is typically considerably lower than their single-cell counterparts. Complex mixtures of cells can be purified by fluorescence activated cell sorting (FACS), magnetic purification, or microfluidic systems prior to subjecting them to these technologies, and recent computational approaches provide the means of deconvolving mixed populations when purification is not feasible or gene expression data from such mixed populations have already been collected (Racle et al., 2017; Newman et al., 2015; Newman et al., 2019; Ahn et al., 2013; Yoshihara et al., 2013; Gong and Szustakowski, 2013; Li et al., 2016b; Becht et al., 2016; Shen-Orr et al., 2010; Zhong et al., 2013; Aran et al., 2017; Quon et al., 2013; Shen-Orr and Gaujoux, 2013; Vallania et al., 2018; Du et al., 2019).

The cornerstone technologies underlying both cancer systems biology and systems immunology are genomic sequencing (WGS and whole exome sequencing, WES) and transcriptomic sequencing (RNA sequencing (RNA-seq)) (Table 1). At its core, cancer is a genetic disease; malignant transformation is the consequence of mutations in tumor suppressor genes and oncogenes (Stratton et al., 2009). WGS and WES have shed light on the contributions of multiple mutations or copy number variations in such genes, and RNA-seq has revealed pathways and signaling networks involved in tumor progression (Creixell et al., 2015; Lawrence et al., 2014; Garraway and Lander, 2013). While the genomes of leukocytes exhibit considerably less variance than those of malignant populations, there are notable exceptions such as the B cell receptor (BCR) and T cell receptor (TCR) present on B and T lymphocytes, respectively. The genomic loci for these receptors undergo rearrangement in order to generate diversity of the antigen-recognition domains in a manner that confers specific immunity against an enormous range of pathogens (Hozumi and Tonegawa, 1976). Elevated expression of a variety of normal proteins, expression of embryonic proteins and antigens, and expression of mutated proteins (neoantigens) all represent targets on tumor cells that can be recognized by BCRs and TCRs to elicit antitumor responses by the immune system. Consequently, cancer systems immunologists have employed targeted amplicon sequencing (typically, of cDNA derived from amplified TCR or BCR mRNA) to evaluate the BCR and TCR repertoires, providing insight into how lymphocytes respond to tumors (Han et al., 2016; Page et al., 2016; Woodsworth et al., 2013; Sims et al., 2016; Linnemann et al., 2013; Jiang et al., 2019; Liu et al., 2018; Chaudhary and Wesemann, 2018; Zhang et al., 2017b). Furthermore, researchers have used WES, frequently in combination with RNA-seq, to identify the range of potential neoantigens expressed by tumor cells as a consequence of their high mutation rates (Garcia-Garijo et al., 2019). Finally, RNA-seq has enabled researchers to identify gene networks and transcriptional programs exploited by tumors to evade anti-tumor immunity, as well as changes in the states of immune cells as they interact with tumors.

While genomic and transcriptomic analyses have been mainstays for Systems Biologists, both cancer biology and immunology have benefitted from epigenetic studies (Egger et al., 2004; Flavahan et al., 2017; Esteller, 2008; Suvà et al., 2013; Li et al., 2013; Schmidl et al., 2018; Busslinger and Tarakhovsky, 2014; Peng et al., 2015; Berdasco and Esteller, 2010; Feinberg and Vogelstein, 1983; Henning et al., 2018). A number of technologies have enabled investigations into the epigenetic control of gene regulation, and by combining these methods with NGS approaches capable of querying the entire genome, researchers have been able to apply systems-level analyses to epigenetics (Table 2). Methylation represents one of the most common epigenetic modifications for silencing transcription (Jones and Takai, 2001), and can be surveyed at the genome level through the use of Whole Genome Bisulfite Sequencing (WGBS) or Reduced Representation Bisulfite Sequencing (RRBS), which use sodium bisulfite to convert unmethylated cytosine residues to uracil while leaving their methylated counterparts (5-methylcytosine) intact (Frommer et al., 1992; Lister et al., 2009; Meissner et al., 2005; Booth et al., 2012; Booth et al., 2014; Yu et al., 2012). To interrogate how specific proteins (e.g. transcription factors) interact with DNA, researchers often use Chromatin Immunoprecipitation (ChIP). In this approach, DNA is crosslinked to proteins with which it is interacting, sheared, and precipitated through the use of antibodies against the protein of interest (Gilmour and Lis, 1985). Combining ChIP with NGS (ChIP-seq), enables the generation of genome-wide maps of DNA binding to proteins of interest (Johnson et al., 2007; Barski et al., 2007). An improved version, known as CUT and RUN, also enables a similar approach to be performed in situ with considerably lower background (Skene and Henikoff, 2017). In immunology, Bisulfite-Seq and ChIP-seq have proven effective tools for uncovering the underpinning epigenetic modifications driving fate decisions and activation states of leukocytes (Henning et al., 2018; Northrup and Zhao, 2011; Zhang et al., 2012; Russ et al., 2014; Abdelsamed et al., 2017). Histone modifications represent some of the most important regulators of cell states (Strahl and Allis, 2000), and ChIP-seq has proven to be one of the most effective technologies for querying such changes. For example, ChIP-seq has been used to define the super-enhancer (SE) landscape in CD4^+^ T cells and identify how polymorphisms in these regions can potentiate risk of autoimmune disease (Vahedi et al., 2015). Similarly, using ChIP-seq to profile a variety of methylation and acetylation patterns of Histone H3, researchers have uncovered the regulatory epigenetic signatures that distinguish the naïve, effector, central memory, and effector memory CD8^+^ T cell subsets (He et al., 2016; Rodriguez et al., 2017; Araki et al., 2009). In cancer biology, ChIP-seq has proven effective for defining differential enhancer signatures in tumor cells (Akhtar-Zaidi et al., 2012). Mutation-independent epigenetic control of tumor suppressors through trimethylation of histone H3 at lysine 4 (H3K4me3) has been uncovered using ChIP-seq approaches (Chen et al., 2015b). Similarly, ChIP-seq has revealed patterns of promoter and enhancer invasion by Myc to drive widespread RNA biogenesis in both tumors and immune cells (Sabò et al., 2014; Lin et al., 2012; Nie et al., 2012).

In addition to approaches that evaluate acetylation, methylation, and protein binding to various loci in the genome, tools to interrogate higher order structure of the genome have recently been developed. Techniques such as DNAse-seq and ATAC-seq (Assay for Transposase Accessible Chromatin) can identify regions of open and closed chromatin (i.e. chromatin accessibility) across the genome (Buenrostro et al., 2013; Boyle et al., 2008; Thurman et al., 2012). These approaches use enzymes to cleave regions of DNA that are not tightly wrapped around nucleosomes, presumably due to active transcription or their occupancy by DNA-binding proteins (e.g. transcription factors). They also enable transcription factor footprinting to identify transcription factor binding sites. Furthermore, by combining these methods with computational approaches, the effects of *cis*- and *trans*-regulatory elements upon gene function can be analyzed. A modified version of these techniques can simultaneously enable Bisulfite-seq on the same sample (methyl-ATAC-seq) (Spektor et al., 2019). Chromatin accessibility analyses have enabled genome-wide characterization and determination of functional implications of such changes in a range of cancers (Corces et al., 2018; Denny et al., 2016), leukocytes (Buenrostro et al., 2018; Shih et al., 2016; Scharer et al., 2017; Sen et al., 2016), and tumor immunology studies (Satpathy et al., 2019; Corces et al., 2016; Philip et al., 2017; Benci et al., 2016). Such approaches have revealed how widespread increases in chromatin accessibility enable transcriptional programs that drive tumor progression and metastasis (Denny et al., 2016). Other studies found that dysfunctional tumor-specific CD8^+^ T cells enter one of two distinct chromatin states that determine whether they can be reprogrammed (Philip et al., 2017).

While regions of open chromatin reveal evidence of transcriptional regulation, higher order chromatin structures also play critical roles in controlling gene expression. Long-range distal elements, such as enhancers, affect gene expression even at distances greater than 1 Mb in linear genome space (Lettice et al., 2003; Bulger and Groudine, 2011; Dekker, 2008). To assess how three-dimensional conformations affect regulation, a variety of technologies have been developed that are capable of querying chromosomal interactions at the genome scale (Davies et al., 2017). These include derivatives of chromosome conformation capture (3C) (Dekker et al., 2002) such as circular chromosome conformation capture (4C) (Zhao et al., 2006; Simonis et al., 2006), chromosome conformation capture carbon copy (5C) (Dostie et al., 2006), NG Capture-C (Hughes et al., 2014; Davies et al., 2016), Hi-C (Lieberman-Aiden et al., 2009), and methods that combine 3C with ChIP such as chromatin interaction analysis by paired-end tag sequencing (ChIA-PET) (Fullwood et al., 2009) and HiChIP (Mumbach et al., 2016; Mumbach et al., 2017). These 3C methodologies are variants of protocols wherein DNA is crosslinked, digested by restriction endonucleases, ligated together, and amplified by PCR to identify regions in close proximity. In particular, Hi-C has enabled mapping of all interactions within the genome at kilobase resolution (Rao et al., 2014). These techniques have informed a variety of studies in both tumor biology and immunology. Earlier studies using fluorescence in situ hybridization (FISH) and 3C originally suggested that interchromosomal interactions between promoters and enhancers on distinct chromosomes can drive immune cell development (Hewitt et al., 2008; Ling et al., 2006), but subsequent high-resolution genome-wide studies using Hi-C failed to confirm the existence of such interactions (Johanson et al., 2018). Recently, as part of the Cancer Genome Atlas (TCGA) studies, enhancer activity has been mapped across nearly 9000 cancer patients combining RNA-seq and Hi-C data to characterize enhancer-gene interactions, and this effort identified a key enhancer of the immunomodulatory protein programmed death ligand 1 (PD-L1) (Chen et al., 2018). Integrating these multiple types of epigenetic signatures with transcriptional datasets should enable the generation of systems level models for transcriptional regulation in both malignant and immune populations during tumor progression. For example, how do genome-wide changes in chromosomal confirmations within immune or malignant cells alter their states of differentiation, and how do heterotypic cellular interactions drive these changes?

### Single-cell sequencing technologies

While light and electron microscopy endowed scientists with the ability to survey biology at the resolution of single cells, perhaps the most influential single-cell technological advance in immunology was the invention of flow cytometry and fluorescence activated cell sorting (FACS) (Fulwyler, 1965; Hulett et al., 1969). These technologies enable scientists to identify and enumerate phenotypically and functionally distinct immune cells, and query the activation states of individual cells in a suspension based on excitation of fluorescent probes (e.g. fluorescently labeled antibodies, fluorescent protein-based genetic reporters, fluorescent DNA intercalating dyes, etc.). In addition to representing a fundamental tool for investigating and identifying the cellular components of the immune system, flow cytometry has been useful clinically. For example, prior to the discovery of the Human Immunodeficiency Virus, it served as the primary means for identifying individuals at risk for AIDS and monitoring their course and response to therapy, based on the relative frequency of circulating CD4 T cells (Lifson and Engleman, 1989). Today, in addition to its use in monitoring the frequencies of various immune cell types in patients with cancers and recipients of organ transplants who are receiving immunosuppressive drugs, flow cytometry is used to characterize malignant cells in blood for clinical diagnosis. By multiplexing these probes, scientists can simultaneously measure tens of markers on individual cells at rates of thousands of cells per second, and recent advances (discussed later) have increased this multiplexing to above 40 markers (Bendall et al., 2012). Due to its ability to survey the states of mixed populations at a single-cell resolution, flow cytometry has facilitated the majority of major discoveries in the field of immunology.

Adaptation of these bulk NGS technologies to single cells has recently led to insights at the single-cell level previously thought to be unattainable. These new technologies were largely enabled by advances in microfluidics and are predicated upon one of three general approaches: (1) single cells are sorted into individual wells of a plate by using conventional FACS, (2) single cells are captured in capture sites of a microfluidic chip, or (3) single cells are captured in emulsion droplets generated in a microfluidic chip. Both microfluidic approaches were derivatives of technologies developed at the turn of the century (Unger et al., 2000; Thorsen et al., 2001; Anna et al., 2003; Thorsen et al., 2002; Tice et al., 2003), wherein precise control of picoliter volumes, aided by advances in soft lithography, is utilized to isolate individual cells and subject them to chemical and enzymatic reactions. These approaches are particularly amenable to DNA sequencing approaches, as reactions involving endonucleases, transposases, and reverse transcriptases as well as PCR can all be performed with exquisite control in these volumes and formats.

The vast majority of bulk NGS approaches have been adapted to these single-cell formats (see Table 1). The primary tradeoff is that while these approaches provide insights into the distinct profiles of individual cells, far fewer sequencing reads can be gathered for a given cell than bulk approaches due to the low abundance of transcripts or DNA within a cell as well as the high costs of sequencing hundreds to tens of thousands of cells at high read depths. Stochasticity, transcriptional bursting, and dropout also add challenges to interpreting single-cell data. Nonetheless, a variety of computational tools have been developed to help account for some of these effects, and the resultant analyses have been transformative for the field of tumor immunology. Some of the single-cell NGS approaches include RNA-seq (scRNA-seq) (Hashimshony et al., 2012; Islam et al., 2011; Shalek et al., 2013; Ramsköld et al., 2012; Tang et al., 2009; Wu et al., 2014; Tang et al., 2010; Tariq et al., 2011; Klein et al., 2015; Macosko et al., 2015; Gierahn et al., 2017; Ziegenhain et al., 2017; Ding et al., 2020; Table 3), genome and exome sequencing (Gawad et al., 2016; Wang et al., 2014a; Navin et al., 2011; Baslan et al., 2012; Xu et al., 2012), nucleus sequencing for RNA or DNA (scNuc-seq) (Wang et al., 2014a; Habib et al., 2016; Habib et al., 2017; Lacar et al., 2016; Hu et al., 2017a), WGBS or RRBS (scBS-seq) (Smallwood et al., 2014; Clark et al., 2017; Guo et al., 2013; Farlik et al., 2015; Angermueller et al., 2016), ChIP-seq (scChIP-seq) (Grosselin et al., 2019; Rotem et al., 2015), ATAC-seq (scATAC-seq) (Satpathy et al., 2019; Cao et al., 2018; Cusanovich et al., 2018; Cusanovich et al., 2015; Buenrostro et al., 2015; Satpathy et al., 2018), and Hi-C (scHi-C) (Ramani et al., 2017; Nagano et al., 2013; Stevens et al., 2017).

The immune system is comprised of a myriad of cell types with distinct functions working in concert to elicit responses against a pathogenic insult. The multitude and diversity of these cell types render bulk sequencing approaches challenging in that the aggregation of reads from the pool of different cell types often masks differences exhibited by particular subsets. Furthermore, changes in the frequencies of cell types are difficult to distinguish from changes in gene expression within those cells. For example, an increase in *IFNG* within a tumor may reflect increased IFN-γ production by T cells already within tumors or an increase in T cell infiltration into the tumor. Recent computational approaches have helped deconvolve immune subsets from bulk RNA-seq data (discussed below), but single-cell sequencing presents an opportunity to accurately quantitate genome-wide changes at the resolution immunologists have become accustomed to from flow cytometry. Applied to tumor immunology, these studies have revealed heterogeneity in both the malignant and hematopoietic compartments, identified novel subsets, aided in reclassification of existing subsets, revealed activation, exhaustion, and suppression states of multiple immune types within tumors, shed light on responses to immunotherapy, and much more.

### Single-cell proteomics

Long before the advent of NGS, immunologists and cancer biologists have been performing low- to moderate-dimensional single cell protein analyses using a variety of platforms such as ELISPOTs, flow cytometry, and various forms of microscopy. More recently, scientists have developed new technologies to achieve higher dimensionality with theoretical ranges reaching the entire proteome. A recent study used confocal immunofluorescence (IF) to image and characterize the subcellular localization of over 12,000 human proteins at the single-cell level and presented the results in an interactive database known as the Cell Atlas (Thul et al., 2017). Using an approach based off of Edman Sequencing and total internal refraction microscopy (TIRF), scientists have demonstrated the ability to identify proteins at zeptomolar concentrations in parallel in a manner that should enable single-cell proteomics (Swaminathan et al., 2018). As single-cell genomic approaches cannot always replace protein level analyses (Latonen et al., 2018), such approaches could greatly enhance our understanding of individual cells.

To assess heterogeneity in leukocyte populations, bioengineers have developed technologies to quantify secreted cytokines at the single-cell level. Using oligo-barcoded antibodies in microfluidic devices, researchers characterized heterogeneity in the secretome of tumor antigen-specific T cells from melanoma patients (Ma et al., 2011). Similarly, a technology based upon single-cell microwells and antibody-coated slides has enabled profiling of the temporal dynamics of T cell cytokine responses at the single-cell level (Han et al., 2012). These researchers also adapted their platform to enable low-cost single-cell RNA sequencing (Gierahn et al., 2017).

One of the most widely adopted technologies used by systems immunologists is mass cytometry (CyTOF) (Bendall et al., 2012). This approach replaces fluorescent tags on antibodies with transition element metal isotopes, to enable measurement of single cells labeled with such antibodies using time-of-flight mass spectrometry (Bandura et al., 2009; Bendall et al., 2011). This technology enables researchers to measure the expression of more than 40 proteins simultaneously on single cells, by eliminating limitations derived from spectral overlap of fluorescent probes. This added dimensionality facilitates a more comprehensive characterization of cellular states or population frequencies, particularly among hematopoietic cells, and has been used to evaluate a wide range of complex processes ranging from hematopoiesis and maturation (Bendall et al., 2014; Good et al., 2019), to antigen-specific responses and vaccine responses (Newell et al., 2013; Newell and Davis, 2014; Swadling et al., 2014), to immune responses to cancer (Hartmann et al., 2019; Good et al., 2018; Lavin et al., 2017; Irish and Doxie, 2014; Chevrier et al., 2017; Simoni et al., 2018; Mistry et al., 2019; Spitzer et al., 2017). By combining immunolabeling with mass tags, mass cytometry has enabled Systems Immunologists to acquire high-dimensional single-cell data for parameters that cannot be measured by single-cell sequencing approaches.

### High-dimensional imaging modalities

With the exception of the peripheral blood, regulation of the immune response relies upon tissue architecture to facilitate homotypic and heterotypic interactions between cells. Secondary lymphoid organs (SLOs, e.g. spleen and lymph nodes) are notable examples wherein local chemokine gradients and tissue architecture enable lymphocytes and APCs to efficiently interact in a manner that orchestrates adaptive immunity (Cyster, 1999; Drayton et al., 2006). Similarly, immune cell locations and interactions within and surrounding tumors are typically not random, and many tumors contain organized lymphoid structures known as tertiary lymphoid structures that are believed to impact clinical outcome (Jones et al., 2016; Joshi et al., 2015; Goc et al., 2013). While the aforementioned single-cell technologies provide the requisite resolution for evaluating cell-cell interactions, they rely upon tissue dissociation and the generation of cell suspensions, which prohibit the evaluation of interactions within the native tissue architecture. Furthermore, the tissue dissociation process itself may affect the states of the cells results of the analyses (van den Brink et al., 2017). Microscopy approaches including light microscopy, fluorescence microscopy, scanning electron microscopy (SEM), transmission electron microscopy (TEM), and variations on these technologies have all enabled clinicians and researchers to investigate tumor-immune interactions in their natural environments, but have been limited in the number of simultaneous parameters that can be measured. As with conventional flow cytometry, spectral overlap limits the number of probes that can be used with fluorescence microscopy. Nonetheless, researchers have used deconvolution approaches with fluorescence confocal imaging to increase the degree multiplexing (Gerner et al., 2012).

Recently, new imaging approaches have been developed that enable increased multiplexing capacity. Following a similar approach as employed with mass cytometry, researchers have combined heavy metal isotope-labeled antibodies to image over 40 parameters simultaneously on histology slides (Angelo et al., 2014; Giesen et al., 2014). In one approach, histology slides are labeled with metal-conjugated antibodies and a layer of tissue is subsequently removed by laser ablation allowing for evaluation using a mass cytometer (Giesen et al., 2014). Another technology, termed multiplexed ion beam imaging (MIBI), uses a focused ion beam to release secondary ions from the antibody-labeled tissue, which are subsequently detected using a magnetic sector mass spectrometer (Angelo et al., 2014). The group that developed MIBI has used it to interrogate tumor-immune interactions in triple-negative breast cancer (Keren et al., 2018).

While mass spectrometry-based approaches enable multiplexing and simultaneous quantitation of all of the parameters at subcellular resolution, they require conjugation of heavy metal isotopes to antibodies as well as access to large and expensive instrumentation. One alternative is to use cycles of conventional immunohistochemistry (IHC) by inactivating dyes or stripping antibodies after imaging and restaining with additional antibodies in a cyclical process. A number of variations of this strategy exist (Gerdes et al., 2013; Schubert et al., 2006; Lin et al., 2015; Tsujikawa et al., 2017; Glass et al., 2009; Wählby et al., 2002), but approaches aimed at minimizing target degradation typically either photobleach the samples or exploit alkaline oxidation to bleach the fluorescence of cyanine dyes after each round of imaging. One drawback to these approaches is that they require repeated rounds of staining. A modified version of this approach, termed CODEX, has been developed wherein oligo-conjugated antibodies are used to stain all antigens at once. Subsequently, fluorophore-labeled complementary oligos to two or three of the antigens are hybridized to the probes, imaged, and removed. Additional cycles are performed enabling highly multiplexed imaging without requiring antibody staining between each cycle (Goltsev et al., 2018).

In addition to immunohistochemistry (IHC) approaches, single molecule RNA fluorescence in situ hybridization (FISH) approaches have been adapted to enable multiplexed imaging of tens to thousands of RNA probes in cells and tissues (Chen et al., 2015a; Lubeck and Cai, 2012; Lubeck et al., 2014; Coskun and Cai, 2016; Levesque and Raj, 2013). In particular, multiplexed error-robust FISH (MERFISH) uses combinatorial barcodes spaced with a Hamming distance of four to enable a high degree of multiplexing while maintaining minimal chances of calling errors (Chen et al., 2015a). Using rolling circle amplification, other groups have developed in situ sequencing approaches capable of evaluating spatial transcript patterns in tissues (Ke et al., 2013; Lee et al., 2014). All these highly multiplexed imaging modalities are designed to enable cancer systems immunologists to investigate how tissue architecture and cell interactions shape immune responses to tumors, and these methods will likely facilitate the development of novel imaging biomarkers for clinical applications in oncology.

### Additional systems-level technologies

While sequencing, cytometry, and imaging technologies are currently the predominant tools employed by systems biologists, a number of other highly multiplexed tools have enhanced systems-level studies of tumor-immune interactions. Alterations in metabolic pathways is a key feature of many cancers (Ward and Thompson, 2012; Warburg et al., 1927). Similarly, immune function is highly impacted by the local metabolic state, and many studies have uncovered important metabolism-mediated tumor-immune interactions that affect tumor progression (Renner et al., 2017). The Human Metabolome Database provides a comprehensive collection of human metabolism data enabling systems-level analyses (Wishart et al., 2007). While most metabolic profiling is performed by conventional forms of MS or nuclear magnetic resonance (NMR) (Holmes et al., 2008; Nicholson et al., 2002), recent advances in imaging mass spectrometry have enabled researchers to profile these pathways directly in tissues (Caprioli, 2016; Kompauer et al., 2017; Sun et al., 2019). Metabolomics has been adopted by systems biologists, who have coined the term ‘Metabonomics’ to refer to the combined outputs of the various metabolomic influences in a multicellular system (Nicholson et al., 2002; Nicholson et al., 2008). Similarly, the field of Glycomics has been transformed by advances in MS and NMR as well as technologies such as glycan microarrays (Song et al., 2011). These technologies have enabled systems-level studies into tumor-immune interactions, and in particular the ability of the immune system to recognize tumor-specific carbohydrate antigens (Satomaa et al., 2009; Liau et al., 2017).

Both tumor cells and immune cells rely heavily upon a variety of soluble proteins for their growth, development, and migration. These collections of secreted proteins, sometimes referred to as the ‘Secretome’ (Tjalsma et al., 2000), have been profiled using a variety of MS-based techniques (Makridakis and Vlahou, 2010; Naba et al., 2012; Meissner et al., 2013). Approaches using antibody arrays and microfluidics have also enabled secretome profiling (Mustafa et al., 2011; Kshitiz et al., 2019). Cytokines and chemokines, in particular, mediate crosstalk between tumors and immune cells and orchestrate immune responses to tumors. To profile these molecules, a number of companies now offer bead-based platforms wherein antibody-labeled beads are incubated with samples and analytes are detected with a secondary antibody, akin to a sandwich ELISA, and quantified on a flow cytometer or dedicated instrument. By performing these assays in solution, multiple analytes can be assayed simultaneously in the same sample, enabling high degrees of multiplexing.

Finally, it is worth noting that while the majority of the technologies discussed above enable characterization of endogenous states, the implementation of genetic screens, particularly those employing the CRISPR/Cas9 system, have made it possible to determine the genetic underpinnings of pathways and phenotypes in a variety of settings (Wang et al., 2014b; Shalem et al., 2014; Doench et al., 2016). These screens have been pioneered in both cancer and immune cells and have revealed previously unappreciated underlying regulatory networks beneath common signaling molecules (Berger et al., 2016; Parnas et al., 2015; Shifrut et al., 2018). In particular, these approaches have been used to interrogate lymphocyte responses to tumors, including in the context of immunotherapy (Shifrut et al., 2018; Dong et al., 2019; Zhou et al., 2014; Pech et al., 2019). A recent study used CRISPR activation (CRISPRa) (Konermann et al., 2015) to activate endogenous genes within tumors as potential neoantigens in a multiplexed fashion that renders the tumors susceptible to immunotherapy (Wang et al., 2019). A number of approaches enable these screens to be applied to single cells (Dixit et al., 2016; Datlinger et al., 2017; Jaitin et al., 2016). Among these approaches is Perturb-seq, which combines multi-locus CRISPR screens with scRNA-seq (Dixit et al., 2016), making it possible to interrogate the effects of higher order interactions that cannot be predicted by the responses to single-gene knockouts. In addition to simply identifying new gene targets, these genome-wide screens should enable systems biologists to discover emergent behaviors of tumor-immune interactions by combining multiple genetic perturbations, cell types, or by performing these screens in vivo.

## Modeling approaches for cancer systems immunology

While often disparate fields, mathematical modeling, computational modeling, informatics tools, and statistical analyses are often inextricably linked, as mathematical modeling (e.g. differential equations) can be aided by computational approaches and bioinformatics tools often rely upon novel or existing mathematical analyses for their implementation. The breadth of modeling approaches and informatics tools in the field of Systems Biology (applied to cancer, the immune system, or both) is too large to review here and has been discussed elsewhere (Germain et al., 2011; Altrock et al., 2015; Meyer and Heiser, 2019; Machado et al., 2011; Norton et al., 2019; Woelke et al., 2010; Stuart and Satija, 2019; Materi and Wishart, 2007). Many modeling approaches, such as Petri Nets (Petri, 1962; Sackmann et al., 2006; Koch, 2015) and Boolean Networks (Thomas, 1973; Saez-Rodriguez et al., 2007; Mai and Liu, 2009; Stoll et al., 2012), have been used extensively to model regulatory and signaling networks within cells (Vieira and Vera-Licona, 2019). While such networks play important roles in governing tumor-immune interactions, the multiscale nature and complexity of immune responses to tumors often renders these approaches challenging to scale up for many applications in cancer systems immunology. Here, we focus on some general principles and tools that are particularly germane to the field of tumor immunology.

Both malignant and immune cells respond to their microenvironments. Factors such as the mechanics of the extracellular matrix (ECM), gradients in cytokines and chemokines, availability of nutrients, and much more drive cells to alter their behavior. Frequently, tumor cells and immune cells influence their own behavior and that of each other by inducing changes in these factors (e.g. immune cells secreting cytokines to recruit and activate more immune cells or cancer cells invading along stiff ECM). To model these elements, mathematicians have frequently turned to systems of ordinary differential equations (ODEs, e.g. for mechanics or chemical reactions) (Ideker, 2001; Bangasser and Odde, 2013) and partial differential equations (PDEs, e.g. for reaction-diffusion analyses) (Turing, 1952; Matzavinos et al., 2004; Khain and Sander, 2006). These models are both intuitive and adaptable and can be solved under many conditions with the assistance of computational approaches. Indeed, such deterministic models have been employed to evaluate tumor-immune interactions (Bellomo et al., 1999; Matzavinos et al., 2004; Owen and Sherratt, 1998). With increasing complexity (e.g. inclusion of many cell types, all producing gradients of multiple soluble factors and responding to them outside the assumptions of steady state conditions), however, analysis of ODEs and PDEs can become cumbersome and computationally impractical, and in such instances, alternate approaches can be more computationally feasible.

A variety of approaches have been used to model competition between immune cells and different clones within tumors. Probabilistic models that utilize evolutionary game theory (EGT) can be amenable to such modeling (Basanta et al., 2012; Pacheco et al., 2014; Stanková et al., 2019; Nowak and Sigmund, 2004; Archetti, 2013; Bellomo and Delitala, 2008). Rule-based approaches have gained popularity for modeling complex systems. Cellular automaton (CA) models can be used to study effects of the microenvironment on tumor growth (Jiao and Torquato, 2011; Gerlee and Anderson, 2007) and immune system homeostasis (Seiden and Celada, 1992; Kaufman et al., 1985). These models place cells within lattices and rely on defined rules to simulate their interactions. Stochasticity (e.g. Brownian motion) can be incorporated into such models as well as the ability to evaluate temporal responses. Perhaps, one of the most powerful modeling approaches in systems biology is agent-based modeling (ABM) (Soheilypour and Mofrad, 2018; Holcombe et al., 2012; Pogson et al., 2008; Odell and Foe, 2008; Drasdo and Höhme, 2005; Ghaffarizadeh et al., 2018; Poleszczuk et al., 2016; An, 2008; Chiacchio et al., 2014). ABM is based upon the interactions of agents, which can be anything from molecules, to cells, to organisms. Their interactions and behaviors are governed by a set of rules, but unlike CA, they are not restricted to a lattice. The agents are autonomous; they can evolve, and their interactions can reveal collective emergent behavior. The ability to simulate interactions between thousands of agents, which could represent a myriad of cell types or molecules, renders this approach particularly amenable to the study of tumor immune interactions (Norton et al., 2019). A number of studies have used ABM to model these interactions during tumor progression (Enderling et al., 2012; Alfonso et al., 2016; Pourhasanzade et al., 2017; Dréau, 2009) or response to immunotherapy (Pappalardo et al., 2011; Gong et al., 2017). PhysiCell is an ABM tool that has been used to interrogate adaptive immune responses to tumors (Ghaffarizadeh et al., 2018; Ozik et al., 2018). In this model, oxygen consumption by cells can result in necrosis and is determined by a normally-distributed parameter representing ‘oncoprotein’ expression. This parameter also affects immunogenicity of a given cell (as an approximation for neoantigen burden) and affects the degree to which immune cells attack the tumor. PhysiCell analyses have revealed how gradients of immunogenicity can ‘trap’ immune cells at the tumor center and allow tumor cells at the outside to escape immune attack and reestablish a tumor (Ozik et al., 2018). Another recent study combined EGT with ABM to model tumor invasion and show how subpopulations within tumors can co-opt distinct macrophage populations to both degrade stroma and suppress immune responses (Gatenbee, 2019).

**Figure 2.**
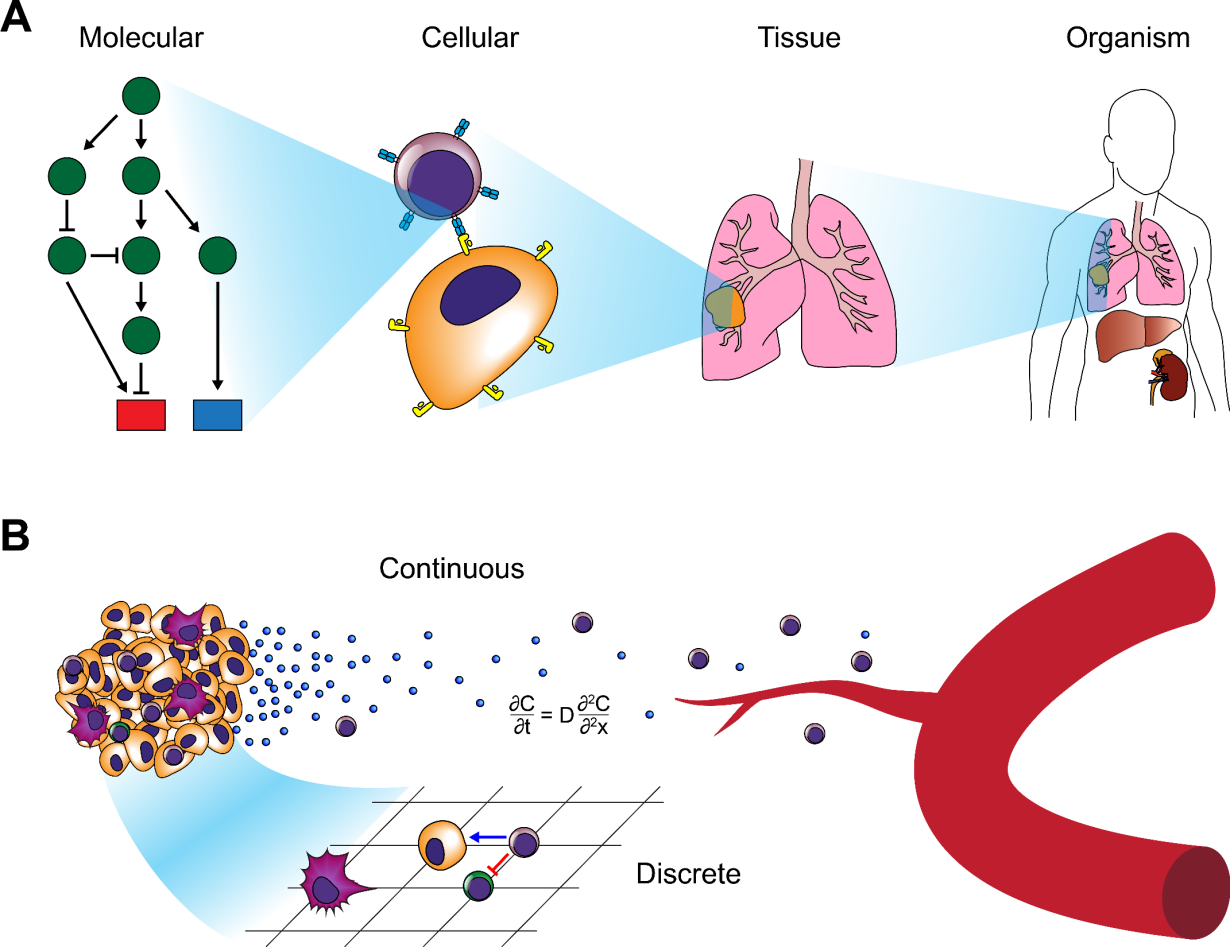
Modeling approaches for cancer systems immunology. (**A**) Modeling approaches used in cancer systems immunology operate within or across multiple scales in order to describe how tumors interact with the immune system. (**B**) Hybrid models, in particular, combine continuous models (e.g. differential equations) with discrete models (e.g. CA or ABM).

One of the most promising aspects of systems biology analyses is their potential to integrate observations across multiple physical and temporal scales to discover emergent behavior that is not discernable from analysis of the individual components. This potential is especially attractive in the tumor setting where orchestration of immune responses occurs across all these levels (Figure 2A). To address these types of problems, systems biologists often use multi-level or hybrid analyses (Norton et al., 2019; Gerlee and Anderson, 2007; Letort et al., 2019; Jeon et al., 2010; Deisboeck et al., 2011; Chamseddine and Rejniak, 2019). Such modeling approaches combine continuous or deterministic models (e.g. ODEs and PDEs) with discrete models such as agent-based models (ABMs). By combining discrete and continuum models, researchers can bridge scales and combine bottom-up approaches that efficiently model cell-cell interactions with rules governed by differential equations spanning larger physical scales to achieve a systems-level understanding of the biology (Figure 2B). For example, combining CA models with PDEs has facilitated investigation of tumor-immune interactions during tumor growth (Mallet and De Pillis, 2006). One hybrid model revealed how spatial and phenotypic heterogeneity can lead to immunosuppression (Wells et al., 2015). These hybrid approaches will enable researchers to bridge existing multivariate datasets and observations to generate more holistic models of tumor immune interactions capable of predicting emergent phenomena that may be difficult to discern with existing experimental approaches.

## Bioinformatics tools for analyzing systems-level data

In addition to the use of computational approaches to develop models of tumor-immune interactions, bioinformaticians have developed a large array of tools for analyzing the data that are produced by the technologies described earlier. While the full extent of these tools is beyond the scope of this review, we highlight a few topics that are particularly relevant to cancer systems immunology.

The reduced cost and increased accessibility of DNA microarrays and NGS have resulted in vast amounts of publicly available human datasets. The Immunological Genome Project (ImmGen) provides transcriptional profiles from all the major immune subsets in mouse and human (Heng et al., 2008; Shay and Kang, 2013). Resources such as InnateDB, ImmuneXpresso, and the Human Immunology Project Consortium also provide large databases of immune expression profiles and interaction networks (Breuer et al., 2013; Kveler et al., 2018; Brusic et al., 2014). Projects such as the Cancer Genome Atlas (TCGA) have collected –omics data from tens of thousands of patients (The Cancer Genome Atlas, 2006). A recent herculean effort by multiple investigators used TCGA data across 33 cancer types and 10,000 tumors to identify six immune subtypes conserved across cancers that can inform outcome predictions and identify regulatory networks independent of tumor type (Thorsson et al., 2018). By performing a pan-cancer meta-analysis of transcriptional profiles in 18,000 patients using a tool called PRECOG, researchers identified specific signatures of distinct leukocyte infiltration that correlates with outcome (Gentles et al., 2015), while another study correlated the antigenicity of tumors with immune responses to characterize the ‘antigenome’ of tumors (Angelova et al., 2015). One of the challenges that arises when analyzing these data is the heterogeneous nature of the tissue collected for these bulk analyses. The sequenced tumors typically contain not only malignant cells but also a variety of stromal and immune populations making it difficult to determine whether observed differences in for example, gene expression or allele frequency, reflect changes within a tumor, changes in other cells, or simply differences in cellular makeup. To address this challenge, bioinformaticians have developed deconvolution algorithms that can approximate cellular content from bulk transcriptomic data (Racle et al., 2017; Newman et al., 2015; Newman et al., 2019; Ahn et al., 2013; Yoshihara et al., 2013; Gong and Szustakowski, 2013; Li et al., 2016b; Becht et al., 2016; Shen-Orr et al., 2010; Zhong et al., 2013; Aran et al., 2017; Quon et al., 2013; Shen-Orr and Gaujoux, 2013; Vallania et al., 2018; Du et al., 2019). CIBERSORTx is capable of not only deconvolving cellular mixtures, but also quantifying cell-type-specific gene expression profiles (Newman et al., 2019). It performs this analysis by developing signature matrices derived from scRNA-seq and FACS sorted bulk RNA-seq datasets. These types of approaches will allow systems biologists to leverage the prodigious amounts of publicly available bulk RNA-seq data to discover how specific immune subsets interact with tumors and influence clinical outcome.

Even with advances in deconvolution, however, scRNA-seq remains one of the most effective methods for evaluating immune profiles within tumors (Shalek et al., 2013; Zilionis et al., 2019; Puram et al., 2017; Azizi et al., 2018; Tirosh et al., 2016; Schelker et al., 2017; Qiu et al., 2019; Zheng et al., 2017). This approach enables interrogation of the immune composition of tumors in an unbiased fashion. The amount of data and diversity of cell types can render interpretation of the datasets challenging, however. Computational biologists have developed a number of tools to analyze these datasets and present the findings in an interpretable fashion (Stegle et al., 2015; Chen et al., 2019a). Typically, expression profiles are clustered in an unsupervised manner and visualized using dimensional reduction techniques (Macosko et al., 2015; Jaitin et al., 2014; Maaten and Hinton, 2008; Žurauskienė and Yau, 2016; Xu and Su, 2015; Guo et al., 2015; Grün et al., 2015; Kiselev et al., 2017; Becht et al., 2019; Levine et al., 2015). Dimensional reduction and visualization approaches such as principal component analysis (PCA) (Pearson, 1901), diffusion maps (Coifman et al., 2005), t-distributed stochastic neighbor embedding (t-SNE) (Maaten and Hinton, 2008), and uniform approximation and projection (UMAP) (McInnes et al., 2018) enable researchers to quickly visualize changes in cell populations. Many ‘all-in-one’ pipelines, such as Seurat, incorporate the normalization, clustering, and visualization algorithms in a single R package to assist in efficient interpretation (Butler et al., 2018; Stuart et al., 2019). A major challenge in scRNA-seq analysis is the occurrence of batch effects. For example, the effects may result in the T cells of one patient clustering more closely with macrophages from the same patient than with T cells from another patient. Such effects are especially prominent when attempting to compare data acquired on different platforms (e.g. 10X Chromium vs. inDrop vs. Fluidigm C1). Furthermore, scRNA-seq captures only a small percentage of the transcripts in any given cell due to undersampling and technical limitationss, resulting in dropout. A variety of techniques have been developed to deal with batch effects and dropout by imputing expression of zero-read genes and defining anchors onto which clusters can be mapped (Stuart et al., 2019; van Dijk et al., 2018). Using a method termed ‘Biscuit’, one approach normalizes and clusters cells simultaneously using co-expression of genes to identify cell types, which are then normalized independently and dropout gene expression is imputed (Azizi et al., 2018; Prabhakaran, 2016). This approach enabled robust comparisons of scRNA-seq datasets across platforms, and was used to demonstrate the diversity of T cell states in human breast tumors.

Single-cell cytometry and imaging technologies also produce large high-parameter datasets whose interpretation has been aided by the development of computational tools. For example, spanning-tree progression analysis of density-normalized events (SPADE) overcomes the limitations of biaxial plots frequently employed in the analysis of conventional flow cytometry to facilitate analysis of mass cytometry data (Bendall et al., 2011; Qiu et al., 2011). This approach uses agglomerative clustering on down-sampled data along with a minimum spanning tree approach to generate a visual representation upon which the original data is displayed. Like many of the mass cytometry analysis tools, SPADE does not rely upon preexisting knowledge and enables an agnostic interpretation of the data. Other methods include viSNE (an adapted version of t-SNE) (Amir et al., 2013), FlowSOM (Van Gassen, 2015), Citrus (Bruggner et al., 2014), PhenoGraph (Levine et al., 2015), X-shift (Samusik et al., 2016). Another tool, Wanderlust, is capable of inferring a trajectory continuum of cell states from mass cytometry data and has been applied to understanding the transitions during B cell development (Bendall et al., 2014). In collaboration with the lab of Garry Nolan, our group developed a tool known as Scaffold Maps that uses manual gating of mass cytometry data to define landmark nodes in combination with force-directed layouts to generate a graphical reference map of the immune system that can be used to compare tissues, species, or other parameters (Spitzer et al., 2015). By augmenting this approach with statistical inference adopted from the significance analysis of microarrays (SAM) (Bair and Tibshirani, 2004), we applied this approach to interrogating cancer immunotherapy in genetically-engineered mouse models of breast cancer and melanoma (Spitzer et al., 2017). These analyses revealed the importance of secondary lymphoid tissues in orchestrating anti-tumor immune responses as well as identified an emergent CD4^+^ T cell population that is a key element of effective immunotherapy. As with mass cytometry, high-parameter imaging modalities require the advent of new analysis tools. A recent study exploring immune infiltration in triple-negative breast cancer patients by MIBI used a deep-learning approach to aid in image segmentation and revealed populational co-occurrence patterns that correlate with prognosis (Keren et al., 2018). Deconvolution approaches have also been applied in combination with multiplexed fluorescence confocal imaging to interrogate immune interactions in entire organs, such as lymph nodes (Gerner et al., 2012). This approach was recently used to demonstrate how autoreactive T cells are regulated by clustering with Tregs and migratory dendritic cells in lymph nodes (Liu et al., 2015). To visualize interactions between immune cells such as T cell interactions with antigen-presenting DCs, researchers have traditionally used two-photon excitation microscopy (Stoll et al., 2002; Mempel et al., 2004; Miller et al., 2002). In a recent study, researchers developed a deep convolution neural network to identify and characterize DC-T cell interactions in fixed tissue samples, thus enabling quantification of these interactions tissues from mice and humans without requiring the use of transgenic animals expressing fluorescent reporters (Liarski et al., 2019). These analyses could have important implications for understanding how tumors interact with the immune system during malignant progression.

## Systems approaches to understanding the roles of specific immune subsets in the tumor immune microenvironment

Studies investigating the immune status of tumors have revealed that the prognosis of patients often strongly correlates with the degree of T cell infiltration into the local tumor microenvironment (TME) (Galon et al., 2006; Binnewies et al., 2018; Gajewski et al., 2013). Immune infiltrates have also served as effective prognostic biomarkers of response to immune checkpoint blockade (ICB) (Herbst et al., 2014; Tumeh et al., 2014). Tumor immunologists frequently refer to tumors as ‘hot’ or ‘cold’ (or ‘deserts’, in extreme cases) to describe the degree of infiltration of immune cells beyond the tumor margin, but these crude epithets fail to capture the breadth of nuance, and presumably prognostic value, that can be extracted using systems biology approaches to interrogate the TME. Furthermore, the TME does not exist in isolation, but rather is the product of constant communication with the entire organism (Egeblad et al., 2010) rendering its analysis particularly amenable to systems approaches. While most systems analyses have focused on immune responses within the primary tumor or peripheral blood, some recent studies have extended their analyses to the TME of metastatic sites such as LNs (Puram et al., 2017; Tirosh et al., 2016; Kim et al., 2020). Further studies are needed to comprehensively interrogate systemic immune responses to metastases, as lymph node metastases can render the systemic immune response permissive to tumor progression (Reticker-Flynn et al., 2020), and the invocation of systemic immunity is required for the efficacy of immunotherapy (Spitzer et al., 2017).

### Macrophages

In the mid-nineteenth century, Rudolf Virchow first recognized that tumors frequently contain leukocytes. He posited that chronic inflammation lies at the origins of tumors (Virchow, 1863; Virchow, 1858), leading Dvorak, 1986, to describe tumors as ‘wounds that do not heal’, a century later. Many of the initial investigations into the TME focused on the role of myeloid cells in promoting tumorigenesis and metastasis (Joyce and Pollard, 2009; Qian and Pollard, 2010; Coussens and Werb, 2002; Engblom et al., 2016). In particular, researchers have focused on macrophages as key instigators of both tumor-promoting inflammation as well as immunosuppression. While macrophages represent essential components of innate immunity due to their capacity to scavenge for microbial pathogens, drive new blood vessel formation, and process and present antigens to lymphocytes, they have long been associated with a range of pathologies including atherosclerosis, cirrhosis, neurodegeneration, and malignancy (Murray and Wynn, 2011). Nearly all solid tumors exhibit evidence of macrophage involvement regardless of the presence of other infiltrating immune types. While tumor-associated macrophages (TAMs) have been reviewed extensively elsewhere (Lewis and Pollard, 2006), we focus here on recent systems biology approaches employed to elucidate their roles in the TME.

Macrophages within tissues are derived from one of two sources: either, they differentiate from circulating bone-marrow-derived monocytes that have extravasated from blood vessels or, in the case of some specialized tissues, they were seeded during development by macrophages derived from the yolk sac or monocytes from the fetal liver (Ginhoux and Guilliams, 2016; Epelman et al., 2014). Langerhans cells of the epidermis (Merad et al., 2002), microglia of the brain (Ginhoux et al., 2010; Ajami et al., 2007; Yona et al., 2013), Kupffer cells of the liver (Yona et al., 2013), and alveolar macrophages of the lungs (Yona et al., 2013; Hashimoto et al., 2013; Guilliams et al., 2013) are all examples of the latter ontogeny and are known as tissue-resident macrophages. Circulating bone-marrow-derived monocytes serve as the other major macrophage source and the primary source after birth. The majority of these monocytes are known as inflammatory or classical monocytes. They express high levels of Ly6C (in mice) and are recruited to sites of inflammation, often through CCR2, where they differentiate into macrophages (although such differentiation typically does not occur during steady state conditions) (Jakubzick et al., 2013; Tsou et al., 2007; Serbina and Pamer, 2006). Ly6C^lo^ nonclassical or patrolling monocytes typically remain within vessels and crawl along the endothelial walls to clean up debris from dying endothelium (Auffray et al., 2007; Carlin et al., 2013).

The majority of TAM research has focused on macrophages derived from inflammatory monocytes and recruited to the TME (Kitamura et al., 2015; Richards et al., 2013). Classically, macrophages have been divided into M1 and M2 macrophages in an attempt to mimic the nomenclature of T helper (Th) cells (Mills et al., 2000). M1 macrophages (or classically activated macrophages) were thought to represent a state of polarization induced by Th1-derived cytokines such as IFN-γ or microbial stimuli (e.g. LPS). They exhibit elevated MHC-II expression and produce nitrous oxide (NO), reactive oxygen species (ROS), TNF-α, and a milieu of inflammatory cytokines (e.g. IL-12, IL-23, IL-6, IL-1, etc.) (Dalton et al., 1993; Mantovani et al., 2004; Atri et al., 1801). In contrast, M2 macrophages (alternatively-activated macrophages) were initially reported to be polarized by Th2 cytokines such as IL-4 and IL-13 that drive alternative macrophage activation (Stein et al., 1992; Doyle et al., 1994; Gordon, 2003). These macrophages express arginase 1, IL-10, CCL17, CCL22, and CCL24 and are generally considered anti-inflammatory and immunosuppressive (Biswas and Mantovani, 2010). Consequently, M1 macrophages have traditionally been associated with anti-tumor effects while M2 macrophages were considered pro-tumor (though M1 ROS production has been suggested to play a role in tumorigenesis) (Kratochvill et al., 2015; Zhang et al., 2013). Over time, it became apparent that the initial nomenclature, derived primarily from in vitro activation experiments, was likely not sufficient to classify the M2 macrophage states, in particular. Distinctions between Th2 cytokine production, IL-10-mediated immunosuppression, and immune complex and TLR stimulation have led to an effort to subset M2 macrophages into three groups (Mantovani et al., 2004).

**Figure 3.**
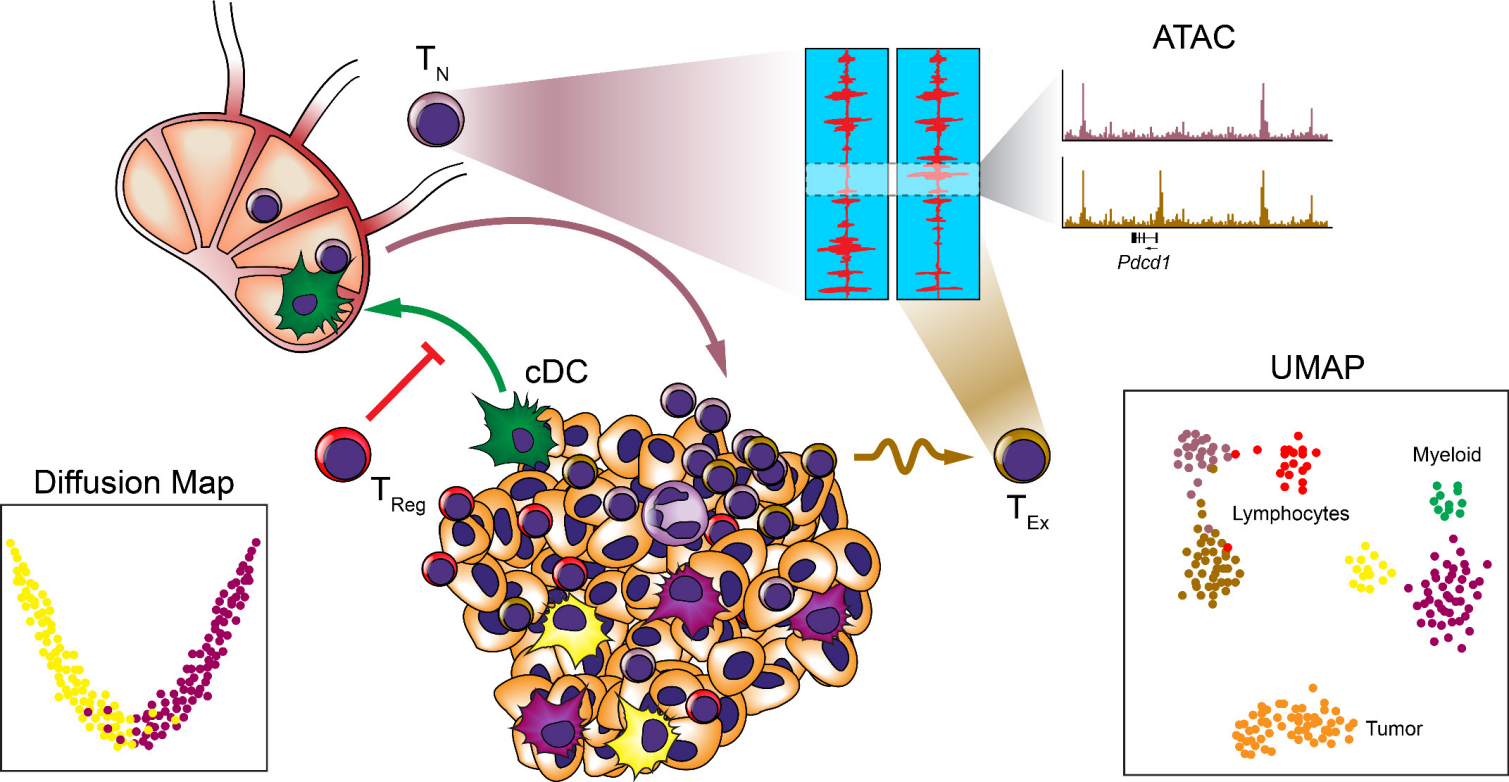
Systems approaches to the TME. Many systems approaches have been applied to analyzing the TME to reveal the cellular makeup and relationships between immune cells at the single-cell level. Studies have revealed heterogeneity among myeloid and lymphocyte populations, trafficking of cDC subsets to the draining LNs, and epigenetic exhaustion profiles of T cells infiltrating tumors following ICB.

Recently, a number of studies that have employed systems approaches have revealed that this binary interpretation of macrophage polarization is vastly oversimplified and fails to capture the diversity of phenotypes exhibited by macrophages (Mosser and Edwards, 2008; Xue et al., 2014; Murray et al., 2014; Martinez and Gordon, 2014; Lavin et al., 2014; Ginhoux et al., 2016; Reinartz et al., 2014; Zhang et al., 2019). One group of investigators interrogated the regulatory landscape of tissue-resident macrophages across a variety of organs by combining RNA-seq, ChIP-seq, and ATAC-seq (Lavin et al., 2014). In this study, the corresponding tissue-resident macrophages from the brain, lungs, liver, spleen, peritoneum, ileum, and colon were purified by FACS, and subjected to these analyses to reveal distinct gene expression, enhancer methylation, and chromatin states depending on their tissue of residence. In accordance with reports describing the considerable plasticity of macrophages, this study also showed that transplanted macrophages could be reprogrammed by their new tissue of residence in a manner that mimics the regulatory landscape of macrophages in those tissues. Seeking to characterize the diversity of macrophage activation states, other investigators performed transcriptional profiling of human macrophages following in vitro activation with a variety of stimuli and found that the cells exhibit a range of distinct states that extend well beyond the conventional M1/M2 polarization axis (Xue et al., 2014). Similarly, TAMs frequently fail to conform to the traditional M1/M2 polarization model (Aras and Zaidi, 2017). In one study that used mass cytometry to profile macrophages and T cells within the tumors of 73 clear cell renal cell carcinoma patients, the authors characterized 17 distinct TAM phenotypes (Chevrier et al., 2017). In lung adenocarcinoma, researchers used both MARS-seq and mass cytometry to reveal that tumor-specific macrophages exhibit distinct profiles from other mononuclear phagocytes, including lung-specific macrophages, conventional macrophages, DCs, and monocytes (Lavin et al., 2017). Characterization of myeloid cell diversity in lung adenocarcinoma using scRNA-seq identified multiple populations of neutrophils, DCs, monocytes, and macrophages that were conserved across patients and species (Zilionis et al., 2019). Of note, while some of the macrophage populations exhibited enrichment for M2 gene signatures, they did not cluster to a singular state and the myeloid cells, in general, exhibit a spectrum of states within tumors. Similarly, another study performed scRNA-seq on 45,000 leukocytes from human breast tumors along with corresponding blood, lymph nodes, and normal breast tissue (Azizi et al., 2018). Using diffusion maps (Coifman et al., 2005), they characterized the heterogeneity in both the T cell and myeloid compartments. In particular, they noted considerable diversity in the myeloid compartments, and surprisingly, found a strong positive correlation between M1 and M2 gene signatures (Figure 3). The three TAM populations that they identified often expressed both gene signatures in the same cells, again suggesting that the original M1/M2 polarization concept does not recapitulate what occurs in vivo, particularly in human tumors. Taken together, these studies suggest that macrophages within tumors do not comport to the theorized discrete M1/M2 polarization states, but rather exist within a spectrum of activation states that even permits concomitant expression of the two profiles. While mixtures of the transcriptional and epigenetic repression by the opposing cytokines IL-4 and IFN-γ may be partially responsible for the mixed phenotypes exhibited in vivo where complex mixtures of cytokines are present (Piccolo et al., 2017), it still remains unclear how such inhibitory programs could result in coexpression of both gene sets within the same cells. Adding to the complexity of TAM characterization, a number of recent studies investigating macrophage ontogeny in the context of tumors have revealed that TAMs are not only derived from bone marrow-derived monocyte precursors, but also tissue-resident macrophages seeded during embryogenesis. A recent study that used parabiosis and congenic mice revealed that TAMs in pancreatic ductal adenocarcinoma are derived from both blood monocytes and tissue-resident macrophages and that these populations serve distinct functions during tumor progression (Zhu et al., 2017). Similarly, lineage-tracing approaches, scRNA-seq, and ATAC-seq have all been used to reveal that both microglia and bone marrow-derived macrophages are involved in gliomas and exhibit distinct profiles and contributions to tumor progression (Bowman et al., 2016; Müller et al., 2017). Through an appreciation of the diversity, ontogeny, and complexity of TAM phenotypes cancer systems biologists are beginning to reveal how the interplay of these populations can facilitate tumor progression.

### Dendritic Cells

Compared to macrophages, dendritic cells (DCs) represent a relative minority of the myeloid cells present within most tumors. Nonetheless, DCs are critical orchestrators of immune responses due to their exquisite capacity to present antigens and license, stimulate, or suppress T cells (Banchereau and Steinman, 1998). They can present antigens in the TME to T cells in situ or in lymph nodes (LNs) by accruing tumor debris that has drained there through the lymphatics or by themselves trafficking from the TME into LNs. Their essential role in choreographing the adaptive immune response across an organism makes them an ideal subject for integrated multi-level systems biology investigations.

Many cells of the DC lineage, including classical DCs (cDCs) and plasmacytoid DCs (pDCs), are derived from a common bone-marrow progenitor, the common DC precursor (CDP), which gives rise to a lineage distinct from other leukocytes (Onai et al., 2007; Naik et al., 2007; Liu et al., 2009; Merad et al., 2013). Traditionally, DCs have been described as belonging to four subsets: cDCs, pDCs, monocyte-derived DCs (MoDCs), and Langerhan cells (LCs), which closely resemble other tissue-resident macrophages in gene expression and phenotype (Satpathy et al., 2012; Eisenbarth, 2019). cDCs, so called as they were the first DCs described (Steinman and Cohn, 1973), are potent antigen presenting cells that efficiently phagocytose antigens and license T cells. Consequently, they play a critical role in facilitating anti- or pro-tumor T cell responses both in the local TME and LNs. cDCs can be further divided into two subsets: cDC1 and cDC2. cDC1 express the chemokine receptor XCR1 (Bachem et al., 2012; Dorner et al., 2009), require the AP-1 transcription factor BATF3 for development (Hildner et al., 2008; Murphy et al., 2013), and are characterized by their ability to cross-present antigens to CD8^+^ T cells. In lymphoid tissues, cDC1s typically express CD8α, while peripheral cDC1s express integrin αE (CD103) in mice (both express BDCA3 in humans) and exhibit the ability to efficiently migrate to LNs to present antigen. Both typically lack CD11b. cDC2 have been primarily associated with activation of CD4^+^ T cells, though both cDC subsets can activate both T cell subsets (Eisenbarth, 2019; Bedoui et al., 2009). While a number of markers have proven useful in delineating DCs subsets, recent unbiased mass cytometry and scRNA-seq studies have added clarity and revealed correlations between DC subsets across anatomical locations and species (Guilliams et al., 2016; Villani et al., 2017; See et al., 2017; Alcántara-Hernández et al., 2017).

It is likely that all of the DC subsets play important roles in tumor progression. pDCs, in particular, have been suggested to play an immunosuppressive role (Demoulin et al., 2013; Sisirak et al., 2012; Mitchell et al., 2018; Zhang et al., 2017c; Labidi-Galy et al., 2011), yet the cDC1 subset is perhaps the best-studied due to its relevance in priming antitumor CD8^+^ T cells, while MoDCs are frequently the most numerous in the TME. cDCs are critical for antitumor T-cell-mediated immunity (Hildner et al., 2008; Fuertes et al., 2011). They exist at the borders of tumors and exhibit prolonged interactions with tumor-specific CD8^+^ T cells, yet they frequently fail to stimulate the T cells to mediate an antitumor effect (Engelhardt et al., 2012; Boissonnas et al., 2013; Broz et al., 2014). Furthermore, migratory CD103^+^ peripheral cDC1s traffic to tumor-draining LNs in a CCR7-dependent fashion, where they interact with tumor-specific T cells and transfer tumor antigens to other LN resident DC subsets, at much higher levels than migratory CD11b^+^ DCs (Roberts et al., 2016; Salmon et al., 2016). FcγR engagement of immune complexes (ICs) by DCs induces their CCR-7 dependent migration to LNs (Clatworthy et al., 2014), and we have shown that the combination of DC adjuvants with tumor-bound allogeneic antibody ICs induce robust anti-tumor T cell responses (Carmi et al., 2015). Vaccines expressing Flt3 ligand (Flt3L), which induces CDP lineage commitment, have been shown to improve ICB efficacy (Curran and Allison, 2009).

Systems-level analyses have helped extend our understanding of cDC functions in tumors. One study, in which mass cytometry was used to evaluate the TME during early- and late-stage tumor development, showed that Flt3L injections vastly increases the numbers of CD103^+^ DCs in the TME (Salmon et al., 2016). Furthermore, these DCs transport antigen to the draining LNs where they augment PD-L1 ICB to elicit anti-tumor T cell responses. Using mass cytometry to profile the evolution of the immune repertoire at an organism-wide level in response to immunotherapy, we found that in the initiation of anti-tumor immune responses, CD103^+^ DCs in the tumor microenvironment exhibit a strong increase in proliferation (Spitzer et al., 2017). Furthermore, the cDC frequencies decrease in the local TME during the therapy initiation phase while increasing in the draining LNs during the rejection phase, suggesting that they may become activated at the tumors and subsequently traffic to LNs to facilitate the generation of anti-tumor T cell responses. Using scRNA-seq, one study found that migration of cDC2s from tumors to the draining LNs is required for priming of CD4^+^ T_conv_ and eliciting a potent antitumor response, but Tregs inhibit their efficacy likely by preventing their migration to the LNs (Figure 3; Binnewies et al., 2019). A recent study combined 10X Chromium with SMART-seq scRNA-seq platforms to investigate the cellular diversity of hepatocellular carcinoma (HCC) and found that the droplet and plate-based approaches identify distinct populations (Zhang et al., 2019). Additionally, they identified a LAMP3^+^ subset of DCs that appeared to migrate from the tumor to draining LNs from their RNA velocity analysis, but these DCs share transcriptional features with both cDC subsets as well as pDCs. This population appears to be inducible from multiple lineages upon stimulation with various DC adjuvants, although it remains unclear whether this subset represents an activation state or a different state of differentiation. These agnostic systems approaches have been particularly useful in understanding these types of complex interactions where DCs are interacting with many different cell types in multiple organs in manners that change considerably over the timecourse of the immune response.

### T cells

While investigations into adaptive immune responses to tumors have long been a major focus of tumor immunologists, the recent successes of immune checkpoint blockade (ICB) and engineered chimeric antigen receptor T cells (CAR-T cells) have stimulated a renewed interest in understanding T cell interactions with tumors. Consequently, many novel systems approaches have been applied to T cells. Due to their ability to recognize tumor-specific antigens and kill tumor cells in an antigen-specific manner, T cells represent one of the most critical components of anti-tumor immune responses. Nonetheless, T cells frequently fail to eliminate tumors likely due to immune evasion (DuPage et al., 2012; Matsushita et al., 2012; Pereira et al., 2017; Sade-Feldman et al., 2017) or suppression (Joshi et al., 2015; Willimsky and Blankenstein, 2005; Pauken and Wherry, 2015) mechanisms of the tumors. Furthermore, while a variety of cells and conditions can promote broad immunosuppression of a host, T cells can become subject to tolerance or exhaustion in a manner that impairs tumor-specific immunity without affecting the ability of the immune system to defend against unrelated pathogens (Schietinger and Greenberg, 2014). It is this latter feature that renders tumors such pernicious adversaries in efforts at both detecting and combatting malignancies.

Systems approaches have aided our understanding of T cell responses to tumors in a variety of contexts. One of the mechanisms by which tumor-specific T cells are thought to become dysfunctional is through processes related to exhaustion. T cell exhaustion, first identified in chronic viral infections (Zajac et al., 1998; Gallimore et al., 1998), is induced by persistent exposure to antigen accompanied by failure to eliminate it (Wherry, 2011). It can take multiple forms and likely evolved as a mechanism to avoid inflammation-induced pathologies in which pathogens cannot be resolved (Blank et al., 2019). It is worth noting that T_Ex_ are not entirely ineffective and exhibit some anti-pathogen efficacy even if unable to eliminate the pathogen. Features of exhaustion include elevated expression of inhibitory receptors (e.g. PD1, Lag3, Tim-3, etc.), reduced cytokine expression, reduced proliferation, altered metabolism, and reduced effector function (McLane et al., 2019). In tumors, T cells often acquire a phenotype that is analogous to exhaustion, although it is unclear whether this dysfunctional state exhibits a similar capacity to be reprogrammed, as is often evidenced in the context of viral infections (Philip and Schietinger, 2019).

Through the use of systems approaches to evaluate the regulatory landscape of tumor-specific T cells, what has become abundantly clear is that, like most T cell states (Zhang et al., 2012; Shih et al., 2016; Yu et al., 2017), the exhausted phenotype of these cells is regulated at an epigenetic level, suggesting that T_Ex_ represent a distinct cell type (Figure 3; Sen et al., 2016). Evaluation of chromatin accessibility landscapes by ATAC-seq has revealed that naïve, effector, memory, and exhausted T cells exhibit profound differences in open chromatin regions (OCR) (Sen et al., 2016). While PD-1 signaling blockade can reinvigorate exhausted T cells, it induces only moderate changes in the OCRs that do not revert the cells to a T_mem_ epigenetic profile, perhaps explaining why the reinvigoration is transient (Pauken et al., 2016). Yet while these epigenetic states persist during PD-1 blockade, as also evidenced through bisulfite sequencing, combining DNA demethylating agents with PD-1 blockade has shown the potential to reverse the exhaustion state and improve antitumor response (Ghoneim et al., 2017). Recent studies combining differential gene expression and OCR analyses between T_mem_ and T_Ex_. suggest that epigenetic control of the exhaustion state is mediated by the transcription factor TOX, which is induced by chronic TCR stimulation, and in particular, calcineurin and NFAT signaling and is responsible for upregulation of inhibitory receptors such as PD1 (Khan et al., 2019; Alfei et al., 2019; Scott et al., 2019). Notably, inhibition of calcineurin signaling following TOX induction by treatment with FK506 or cyclosporin A did not ablate TOX expression or the T_Ex_ phenotype, suggesting that once induced, TOX expression remains stable (Khan et al., 2019). Augmenting these studies, scRNAs-eq has revealed similar TOX-driven phenotypes in the context of chronic viral infection (Yao et al., 2019). Similarly, by combining transcriptional analysis with genome-wide H3K4me3 and H3K27me3 analyses, researchers identified a critical role for the NR4A1 transcription factor in instilling the T_Ex_ state by inhibiting binding of the AP-1 transcription factor to binding sites of effector-related genes (Liu et al., 2019). These effects extend to cell therapies, where genetic ablation of NR4A genes can prevent exhaustion in CAR-T cells and improve tumor clearance (Chen et al., 2019b). Another CAR-T study found similar effects with ablation of *TOX* and *TOX2* and suggested that TOX and NR4A positively regulate each other (Seo et al., 2019).

Studies interrogating the stability of T cell dysfunction and exhaustion in the context of viral infection, autoimmunity, and malignancy have identified a number of states, some of which are defined by their inherent reversibility and others by their proliferative or progenitor capacity, although they are frequently referred to as reprogrammable-non-reprogrammable, partial-complete, or progenitor-terminal exhaustion (Philip et al., 2017; McLane et al., 2019; Schietinger et al., 2016; Long et al., 2016; Blackburn et al., 2008; Paley et al., 2012; Im et al., 2016). Progenitor T_Ex_, or less-differentiated T_Ex_, express high levels of TCF-1 or T-bet and exhibit the capacity to replenish the terminally differentiated T_Ex_ (Paley et al., 2012; Im et al., 2016; Wu et al., 2016; Utzschneider et al., 2016). Of note, these subsets typically respond quite differently upon treatment with PD-1 blockade with the progenitors expanding during therapy. As these states appear to be largely regulated at an epigenetic level, ATAC-seq has been used to reveal distinct chromatin states that distinguish dysfunctional tumor-specific CD8^+^ T cells that can be recovered from those that are resistant to reprogramming (Philip et al., 2017). Additional technologies have facilitated systems analyses of T cell exhaustion. A recent droplet-based ATAC-seq protocol that enables massively parallel scATAC-seq profiling was used to interrogate T cell exhaustion in tumors in the context of ICB (Satpathy et al., 2019). The results revealed a massive expansion of T_Ex_ in patients post treatment with PD1 blockade. The results further revealed differences in OCRs corresponding to intermediate and terminally-exhausted T_Ex_ as well as a shared regulatory program between T_Ex_ and T_fh_. A recent study that combined MARS-seq with single-cell TCR sequencing in melanoma patients to evaluate clonality of dysfunctional T cells revealed that the transcriptional profile of T cells forms a gradient of states exhibiting a range of profiles from highly cytotoxic to highly dysfunctional with shared clones occupying multiple transitional states of dysfunction (Li et al., 2019) Surprisingly, although T_Ex_ are thought to have a reduced proliferative capacity, the most proliferative T cells (by Ki67 and clonal expansion) were the dysfunctional T cells.

In addition to exhaustion, systems analyses of the TME have revealed a variety of other T cell features. By combining scRNA-seq with TCR sequencing, one group of investigators identified specific subsets of CD8^+^ T cells and Tregs that are enriched in hepatocellular carcinoma compared to other tissues and identified a gene, LAYN, that inhibits IFN-γ production (Zheng et al., 2017). The same investigators used this approach to characterize fate decisions in tumors, revealing a variety of relationships between various T cell subsets in the TME (Zhang et al., 2018; Guo et al., 2018). Studies interrogating the tumor specificity of tumor-infiltrating T cells by TCR sequencing (Scheper et al., 2019) and mass cytometry (Simoni et al., 2018) have revealed that many T cells within the TME do not actually recognize tumor antigens. In breast cancer, researchers used scRNA-seq to reveal that T cells with transcriptional profiles analogous to resident memory T cells (T_RM_) can be found within tumors and correlate with improved prognosis (Savas et al., 2018). Using scRNA-seq, ATAC-seq, and TCR-seq to query ICB responsiveness in melanoma patients revealed a variety of T cell-related factors that correspond to ICB efficacy including TCF7 expression on tumors and CD39 blockade (Sade-Feldman et al., 2018). An additional study also suggests that T cells exist along a continuum of differentiation states evidenced by scRNA-seq analyses and that tumor-involved Tregs may exhibit more heterogeneity than previously appreciated (Azizi et al., 2018). In agreement with scRNA-seq reports, a mass cytometry approach found that the lung cancer TME is enriched for T cells that are dysfunctional or regulatory (Lavin et al., 2017).

### B cells

While far fewer studies have interrogated the roles of B cells in tumor progression and antitumor immunity than T cells, tumor-infiltrating B cells can be found in most solid malignancies and are readily amenable to the same types of systems analyses. The primary function of B cells is to orchestrate the humoral immune response through the production of antibodies, but there exist a number of different subsets of B cells that have additional functions (Allman and Pillai, 2008). Some B cells can elicit immunosuppressive activity, including in the context of malignancy, through a variety of mechanisms including expression of PD-L1 and production of IL-10, IL-35, and TGF-β leading some to refer to this subset as regulatory B cells (Bregs) in analogy to Tregs (Schioppa et al., 2011; Yanaba et al., 2008; Rosser and Mauri, 2015; DiLillo et al., 2010; Khan et al., 2015; Olkhanud et al., 2011; Huang et al., 2017). Production of cytokines from B cell subsets such as Bregs, B10 cells, or B1 cells can induce a myriad of effects, including repolarization of macrophage populations, induction of Tregs, or activation of survival signals within tumor cells themselves (Wong et al., 2010; Mielle et al., 2018; Ammirante et al., 2010). Furthermore, B cells can promote lymphangiogenesis (Angeli et al., 2006), which, in turn, can enable trafficking of DCs or tumor cells themselves from primary tumors to draining LNs (Roberts et al., 2016; Binnewies et al., 2019; Stacker et al., 2014; Tammela and Alitalo, 2010). Consequently, B cells have frequently been associated with promoting tumorigenesis and metastasis (de Visser et al., 2005; Shah et al., 2005; Qin et al., 1998; Affara et al., 2014; Brodt and Gordon, 1982). These immunosuppressive mechanisms typically occur in a manner that does not require antigen recognition by the BCR (Shah et al., 2005). Yet other studies have suggested that the production of antibodies themselves may promote tumor progression and metastasis by activation of Fc Receptors (FcRs) on myeloid cells, induction of tumor-intrinsic signaling through activation of surface receptors, promotion of pro-inflammatory granulocyte responses, promotion of angiogenesis, and suppression of cellular immunity (Qin et al., 1998; Pucci et al., 2016; Gu et al., 2019; Andreu et al., 2010; Barbera-Guillem et al., 1999; Nyhus et al., 2001; Tan and Coussens, 2007).

Although B cells have the potential to elicit these tumor-promoting effects, they have also been implicated as key regulators of antitumor immunity in the context of immunotherapy. Tumors often acquire tertiary lymphoid structures (TLSs), which closely resemble lymphoid follicles, containing T cells, B cells, FDCs, T_fh_-like cells, fibroblast reticular cells (FRCs), and even high endothial venules (HEVs) (Jones et al., 2016; Goc et al., 2013; Dieu-Nosjean et al., 2014; Colbeck et al., 2017). Although TLSs can serve as a source of protumoral Tregs (Joshi et al., 2015), their presence in tumors has classically correlated with improved survival in a variety of cancers (Colbeck et al., 2017; Ladányi et al., 2007; Dieu-Nosjean et al., 2008; Pitzalis et al., 2014). Like SLOs, these TLSs contain germinal centers (GCs) where B cells can proliferate and undergo class switching and somatic hypermutation (GeurtsvanKessel et al., 2009; Perros et al., 2012; Neyt et al., 2012), and the presence and differentiation of B cells, in particular, within GCs correlates with improved prognosis (Germain et al., 2014). Thus, activation of humoral responses within TLS can improve responses, particularly in the context of immunotherapy (Germain et al., 2014; Helmink et al., 2020; Montfort et al., 2017; Sautès-Fridman et al., 2016; Cottrell et al., 2018; Sautès-Fridman et al., 2019). Furthermore, while FcR binding of tumor-immune complexes has been suggested to promote tumor progression (Andreu et al., 2010), we have found that combining tumor-binding antibodies with DC adjuvants and CD40 agonists results in extremely potent anti-tumor responses in a manner that is FcR-dependent (Spitzer et al., 2017; Carmi et al., 2015; Carmi et al., 2016).

Given the importance of B cells in both tumor progression and antitumor responses, researchers have begun to apply a variety of systems-level approaches to elucidate their roles in malignancy. While the use of TCR repertoire profiling is discussed in detail in the following section, analogous approaches have been developed for BCRs and employed in the context of malignancy to identify the sequences of tumor-reactive antibodies and identify their cognate antigens (Katoh et al., 2017). Building upon their TRUST algorithm developed to identify TCR CDR3 sequences from bulk RNA-seq data (Li et al., 2016a), researchers adapted their approach to BCRs to evaluate humoral responses in TCGA data across 32 cancer types (Hu et al., 2019). They characterized broad clonal expansion across malignancies and suggested roles for subclass switching in conjunction with defective ADCC. The roles that these alterations play in tumor progression or anti-tumor immune responses, however, remain to be clarified. Similarly, as part of their characterization of six distinct conserved immune subtypes using TCGA data, researchers employed immunoglobulin heavy chain repertoire profiling using V’DJer (Mose et al., 2016) on bulk RNA-seq data. This analysis revealed high variance of IgH diversity depending on immune subtype with the IFN-γ and TGFβ dominated subtypes exhibiting high variance and the ‘lymphocyte depleted’ and ‘immunologically quiet’ subtypes exhibiting considerably lower diversity.

To understand the role of TLS B cells during immunotherapy, one group combined bulk RNA-seq of melanoma and renal cell carcinoma (RCC) responders and non-responders with deconvolution algorithms to identify a strong enrichment in B cell signatures in responders. They further analyzed the BCRs of these patients from the bulk RNA-seq and found increases in both clonal diversity and expansion of individual clones in responders, suggesting antigen-specific humoral responses. Finally, using mass cytometry, they demonstrated an enrichment for memory B cells, plasma cells, and GC B cells specifically within the tumors of responders, suggesting a role for B cell activation and humoral responses in patients benefiting from ICB (Helmink et al., 2020). Other researchers combined novel GEMMs with BCR sequencing and scRNA-seq to identify a role for T_fh_-induced B cells in promoting ICB responses (Hollern et al., 2019). While not as common as existing T cell analyses, these types of multimodal systems approaches to understanding the involvement of B cells in malignancy and associated therapeutic responses will be critical for uncovering the role that these lymphocytes play in tumor progression and treatment.

### NK cells, non-canonical APCs, and other myeloid cells

Although T cells, B cells, TAMS, and DCs have been the subject of the majority of systems analyses of the TME, other lymphocytes, myeloid cells, and non-hematopoietic APCs play important roles in tumor progression. Similarly, the importance of NK cells and other innate lymphoid cells (ILCs) (Cerwenka and Lanier, 2001; Wu and Lanier, 2003; Marcus et al., 2014; Malmberg et al., 2017; Hsu et al., 2018; Bruchard and Ghiringhelli, 2019) and PMNs (Szczerba et al., 2019; Fridlender et al., 2009; Nozawa et al., 2006; Piccard et al., 2012; Eruslanov et al., 2014; Singhal et al., 2016) in tumor progression have been well documented but are only beginning to be explored at a systems level (Zilionis et al., 2019; Horowitz et al., 2013). In addition to DCs, macrophages, and B cells, there exist a number of non-professional APCs capable of inducing tolerance (Turley et al., 2010). Many of these cells, such as lymphatic endothelial cells and fibroblastic reticular cells, exist in or near SLOs and can induce tolerance or immunosuppression, including in the context of tumors (Cohen et al., 2010; Nichols et al., 2007; Lund et al., 2012; Lund et al., 2016; Swartz, 2014; Fletcher et al., 2010; Fletcher et al., 2015; Lee et al., 2007). The functional relevance of these cells in the induction of tumor-specific immune tolerance is in its nascent days of exploration, and systems approaches will certainly aid in advancing our understanding of their role in tumor progression.

For over a decade there has been interest in a group of cells frequently associated with tumors referred to as ‘myeloid derived suppressor cells’ (MDSCs). This nomenclature was based upon expression of the integrin subunit αM (CD11b) and staining (in mice) for a marker known as ‘Gr-1’ with an antibody that exhibits a high affinity for Ly-6G and weak affinity for Ly-6C (Gabrilovich et al., 2007). It should be noted that CD11b, Ly-6G, and Ly-6C are expressed on a wide range of immune cells at various levels including monocytes, neutrophils (PMNs), macrophages, DCs, NK cells, and even T cells. Numerous studies, including our own, have revealed that cells bearing these markers can promote tumor progression, metastasis, or suppression of T cell immunity (Reticker-Flynn and Bhatia, 2015; Gabrilovich, 2017). Nonetheless, expression of these markers, on their own, does not confer immune suppressive function. Indeed, anti-Gr-1 antibodies are frequently used to deplete neutrophils, although such antibodies also deplete some monocytes and T cells due to their Ly-6C expression (Daley et al., 2008; Faget et al., 2018). Furthermore, many studies have demonstrated the immune-suppressive and tumor-promoting capacities of PMNs (Fridlender et al., 2009; Piccard et al., 2012), monocytes (Qian et al., 2011; Jung et al., 2017), macrophages (Noy and Pollard, 2014), and DCs (Kenkel et al., 2017; Shurin et al., 2013) in tumors, making it unclear whether MDSCs represent a distinct activation state or lineage from those cell types. In an attempt to clarify the nature of this population, researchers have further divided MDSCs into granulocytic and monocytic lineages, but acknowledge that the differentiating markers have no impact on the suppressive function of these cells (Youn et al., 2008). Analogous subsets have been described in humans and their identification also relies upon markers shared by many known hematopoietic subsets (Bronte et al., 2016; Mandruzzato et al., 2016). Reliance upon these nonspecific markers has resulted in a concerning number of publications claiming MDSC classification without functionally validating the suppressive capacity of the cells. Functional analysis of sorted populations does not prove that the cells of interest are distinct from other myeloid populations (e.g. monocytes, macrophages, DCs, or neutrophils), but is capable of determining whether some cells in the sorted population have suppressive capacity (Bronte et al., 2016).

Systems biology approaches provide a more comprehensive agnostic approach toward evaluating the myeloid repertoire, as has been discussed above. One study purports to identify a specific marker, LOX-1, of PMN-MDSCs by bulk RNA-seq, though this marker is only upregulated in human PMN-MDSCs (Condamine et al., 2016). Separation of the PMN-MDSC population from PMNs, however, was performed by density centrifugation, which results in mixed populations, highlighting the inadequacy of bulk analysis approaches for populations without unique markers. While LOX-1^+^ cells exhibit an increased capacity to suppress T cell responses, it is unclear whether this is a feature of all LOX-1^+^ cells or a subset and whether the marker represents a distinct lineage rather than an activation or maturation state of PMNs. Furthermore, genetic ablation of the gene (*Olr1*) in mice had no impact on the suppressive function of the cells in vitro nor growth of tumors in wild-type mice reconstituted with *Olr1^-/-^* bone marrow following lethal irradiation. In contrast to bulk approaches, single-cell analyses such as scRNA-seq are particularly useful for identifying distinguishing features of individual cells, evaluating heterogeneity of populations, and reconstructing lineage relationships between populations. Indeed, when unbiased clustering of systems level single-cell data has been performed under a myriad of conditions, none of the studies report the existence of distinct MDSC clusters (Spitzer et al., 2017; Puram et al., 2017; Azizi et al., 2018; Tirosh et al., 2016; Binnewies et al., 2019; Sade-Feldman et al., 2018). In one recent exception, researchers labeled a myeloid cluster ‘MDSC-like macrophages’ due to their expression of S100A family genes, but the suppressive capacity of these cells was not evaluated. Furthermore, these cells fell along a continuum with other macrophages on diffusion maps and did not distinctly segregate from other myeloid populations in the UMAP projections. Current transcriptional, epigenetic, and proteomic analyses do not support the notion that MDSCs represent a singular or dual lineage(s) or distinct differentiation states. Most likely, they represent a heterogeneous plastic phenotypic state of neutrophils, monocytes, macrophages, and their precursors, some of which exhibit immune suppressive capacity. Thus, it is best to use caution when employing a nomenclature that ascribes function based solely upon unrelated marker expression.

### T cell receptor repertoires and neoantigens: understanding and harnessing tumor-immune specificity

While the generation of effective naturally occurring or therapeutically induced immune responses typically requires the involvement of many immune cell types, T cells are frequently the most critical element of these responses. Patient prognosis often correlates with the degree of T cell infiltration into tumors (Galon et al., 2006; Zhang et al., 2003), and adoptive transfer of ex vivo stimulated autologous tumor infiltrating lymphocytes remains one of the most effective tumor immunotherapies (Rosenberg et al., 2008; Rosenberg et al., 1988). Similarly, CAR-T therapy involves engineering T cells to directly recognize tumors, and ICB works by stimulating or reinvigorating tumor-reactive T cells. A critical factor explaining the efficacy of T cell responses to tumors is the ability of the TCR to distinguish malignant from healthy tissue by its overexpression of normal antigens, re-expression of embryonic antigens, or expression of neoantigens (mutated proteins expressed only by the malignant cells). Neoantigens are frequently genomic in origin, resulting from point mutations, indels, or translocations, but can also arise from post-translational modifications such as phosphorylation or glycosylation (Bräunlein and Krackhardt, 1702; Cobbold et al., 2013; Zarling et al., 2006). Some cancers may escape T cell surveillance by reducing or eliminating expression of these neoantigens in a process known as ‘immunoediting’ (DuPage et al., 2012; Matsushita et al., 2012; Schreiber et al., 2011), while others escape by downregulating MHC-I presentation (Pereira et al., 2017; Sade-Feldman et al., 2017; Gettinger et al., 2017; Zaretsky et al., 2016; Sahin et al., 2017). Furthermore, efficacy of ICB is correlated with neoantigen burden for a variety of cancers (Yarchoan et al., 2017; Snyder et al., 2014; Carbone et al., 2017; Rizvi et al., 2015a; Galsky et al., 2017; McGranahan et al., 2016), and tumor mutational burden (TMB) may be a better predictor of ICB response than PD-L1-positive staining in some instances (Hellmann et al., 2018). Furthermore, the degree to which neoantigens are shared across tumor clones also correlates with ICB efficacy (McGranahan et al., 2016). Consequently, bioinformaticians and systems biologists have developed an array of tools and approaches for predicting and quantifying neoantigens as well as mapping T cell clonality and neoantigen recognition (Liu and Mardis, 2017).

As effective anti-tumor immune responses typically involve T cell recognition of neoantigens, approaches have been developed to vaccinate patients against the neoantigens of their tumors. Early attempts at cancer vaccines and cell therapies did not rely upon a priori knowledge of the specific neoantigens of the tumors (Fong and Engleman, 2000; Rosenberg and Restifo, 2015). Building off initial proof-of-principal studies in mice (Mandelboim et al., 1994; Mandelboim et al., 1995; Castle et al., 2012), a variety of strategies have recently been designed to specifically target the neoantigens of patient tumors (Schumacher and Schreiber, 2015; Hacohen et al., 2013). The existence of mutations within tumors does not mean that those mutations will serve as neoantigens. In order for a given mutation to result in T cell recognition, it must be in a protein coding region, be non-synonymous, be in a gene that is expressed by the tumor cells, be maintained following proteasomal degradation, result in a peptide that can be loaded onto the specific MHC-I molecules of that patient, and, of course, be recognized by the TCR of a T cell that can enter the tumor. A variety of tools have been developed to use WES data (often combined with RNA-seq data) to predict potential neoantigens in a tumor (Bjerregaard et al., 2017; Lundegaard et al., 2008; Gowthaman et al., 2010; Rajasagi et al., 2014; Duan et al., 2014; Hundal et al., 2016; Łuksza et al., 2017). Additionally, MS has been used to characterize the peptidome of tumors to identify neoantigens directly, frequently by affinity purification of HLA molecules and MS analysis of eluted HLA-bound peptides (Creech, 2018; Cox et al., 1994; Pritchard et al., 2015; Bassani-Sternberg et al., 2010). Using these types of tools, researchers have begun testing pipelines for developing personalized neoantigen vaccines in humans (Sahin et al., 2017; Ott et al., 2017; Carreno et al., 2015; Hu et al., 2017b). These approaches typically begin with excision of tumors and WES to identify candidate neoantigen peptides. In one case, long peptides (15 to 30 amino acids) were synthesized containing the top candidate peptides and administered to patients in conjunction with immune adjuvants in multiple priming and boosting injections. Use of the long peptides was chosen to enable elicitation of both CD4^+^ and CD8^+^ T cell responses. To improve HLA-binding prediction, the same researchers used an LC-MS/MS approach wherein peptides were eluted from single HLA allele-expressing cell lines and subjected to MS resulting in the identification of 24,000 peptides and their cognate HLA class I molecules (Abelin et al., 2017). Furthermore, using this database of peptide-MHC interactions, they trained a neural network algorithm to predict peptide loading with a higher degree of accuracy than preexisting binding affinity-based approaches. The initial neoantigen vaccine trials have now been extended to glioblastoma, a notoriously immune cold tumor, and appear to generate antigen-specific T cells and increases in tumor infiltration by the T cells (Keskin et al., 2019).

In addition to vaccine design, systems approaches to neoantigen identification have been employed for TIL therapy. Following analogous approaches as used for vaccines, researchers identified candidate peptides based on WES of 75 patients with GI cancers. They then screened TIL cultures by ELISPOT for reactivity in response to autologous DCs pulsed with the peptides or transfected with minigenes harboring the mutations. By subsequently sequencing TCRs of T cells responding to particular neoantigens they were then able to transduce new T cells with these TCRs to enable greater T cell expansion and the ability to generate personalized T cell therapies (Parkhurst et al., 2019). These types of approaches highlight the potential benefits that systems biology can bring to patient care and personalized medicine. Only by taking advantage of the synergies of high-throughput technologies, novel bioinformatics approaches, mathematical models, and the ability to monitor and predict immune responses across an entire patient can such an approach be rendered feasible and uncover new avenues for patient care and tumor immunotherapy.

While antitumor T cell-mediated immunity is typically predicated upon recognition of tumor-specific antigens, it also requires a TCR repertoire capable of recognizing those epitopes. The profound diversity of the TCR is not germline encoded but rather is the consequence of V(D)J rearrangement that results in the generation of more possible sequences than there are T cells within an individual (Arstila et al., 1999). While conventional T cells recognize only specific antigens, the TCR is relatively cross-reactive compared to antibodies. One elegant systems-level study used yeast display of peptide-MHC constructs to reveal that a given TCR is capable of recognizing hundreds of distinct peptides provided that they contain specific ‘hot spots’ where the TCR contacts the complex (Birnbaum et al., 2014). The peptides shared among a cross-reactive TCR bear many similarities, permitting researchers to predict potential naturally-occurring ligands given a TCR sequence. The group that developed this approach subsequently applied it to CRC by sequencing patient tumors and TCRs to determine peptide-MHC ligands for expanded TILs (Gee et al., 2018). Epitope prediction revealed TCR-specificity including multiple TCRs that recognized non-mutated self-antigens. Nonetheless, prediction of TCR ligands from their sequences remains one of the holy grails of immunology. To address this need, researchers developed GLIPH, an algorithm capable of grouping TCRs based on predicted shared epitopes using similarity of the CDR3 regions and demonstrated that they could predict shared binding partners across individuals (Glanville et al., 2017). Another study combined MHC tetramer selection with single-cell TCR sequencing to develop an algorithm, TCRdist, that also groups TCRs of related specificities (Dash et al., 2017). Such approaches might help researchers understand whether distinct clones in a patient recognize similar antigens, perhaps present at different tumor sites or even across different patients with similar malignancies. Cancer systems immunologists could exploit existing TCR sequencing data to determine the antigens most frequently recognized by T cells in patients responding to ICB, for example.

In addition to identifying epitopes, understanding the clonal evolution of the T cell response as well as the phenotypes of T cells bearing TCRs of a particular specificity can be highly informative when considering the immune responses to tumors. To investigate the relationships between functions of T cells and their TCR specificity, researchers developed a single-cell sequencing approach for T cells that combines targeted RNA-seq with TCRα and TCRβ gene sequencing (Han et al., 2014). Applying their approach to CRC, they found expanded clones within the tumors that shared specificity and were absent from adjacent normal tissue. Similarly, other researchers combined scATAC-seq with TCR-seq to evaluate clonal relationships with epigenetic profiles and applied it to patients with cutaneous T cell lymphoma to identify differences in the regulatory pathways between normal and leukemic T cells (Satpathy et al., 2018). In a recent study, this same group combined single-cell TCR and RNA sequencing on nearly 80,000 cells from patients with basal and squamous cell carcinomas before and after ICB treatment with anti-PD1 (Yost et al., 2019). By comparing clonotypes with phenotypes, they showed that exhausted and effector CD8^+^ T cells in tumors shared clonotypes with memory T cells but not with each other, suggesting that these distinct states are not shared between the same T cell clones. Even more notably, they found considerable expansion of T_Ex_ post-treatment that did not exist before therapy, with only minor contributions from pre-existing *TCF7*^+^ clones. These clones can be found within the blood at much lower percentages. In contrast with conventional wisdom suggesting that ICB works by reinvigorating existing T cells within the TME, these results suggest that anti-PD1 stimulates recruitment of new tumor-specific T cells to the TME and are in agreement with our own findings that trafficking of lymphocytes from SLOs is required for effective immunotherapy (Spitzer et al., 2017). While this approach cannot rule out the existence of rare T cell clones in the TME prior to treatment that exist below the sequencing depth and expand following treatment, the data suggest this is unlikely and future complementary systems-level studies interrogating clonal trafficking could help shed light on this question.

Nonetheless, while these T_Ex_ clones that are tumor-specific can be found within blood of treated patients and recruitment of T cells from SLOs is important for treatment efficacy, some of these effects may be transient, and it remains unclear which T cell populations are the most critical for effective immunotherapy. It is possible that T_mem_ and cells from both T_Ex_ states within the TME and extratumoral tissues play important roles during immunotherapy. It is important to note that autologous transplant of ex vivo expanded tumor-infiltrating lymphocytes (TIL therapy) has proven effective, and even curative, in a number of malignancies including melanoma, breast cancer, and multiple GI cancers (Rosenberg et al., 1988; Rosenberg and Restifo, 2015; Dudley et al., 2002; Restifo et al., 2012; Tran et al., 2014; Tran et al., 2016; Zacharakis et al., 2018). Moreover, in the case of melanoma, some patients who have failed ICB still benefit from TIL therapy (Sarnaik et al., 2020; Rohaan et al., 2018). These successes highlight the fact that there exist tumor-specific T cells within tumors that can be expanded and used to eliminate tumors. Thus, it is unclear whether these T cells reflect exhausted or dysfunctional T cells whose state is reversible in the context of ex vivo expansion with or without engineering or whether there exist a minority subset of tumor-specific T cells that are not in any state of exhaustion and can be expanded and act as T_Eff_ and T_mem_ once reinfused. In either case, it is quite reasonable to expect that ICB and TIL therapy have highly distinct effects on the nature of the T cells eliciting the anti-tumor immunity. A systems-level characterization of TIL approaches might help shed light on the states of the expanded T cells, whether they were derived from progenitor T_Ex_ or other T cell subsets, and to what extent the ex vivo processing is capable of reprogramming them.

### The microbiome and its effects on tumor immunity

Microbiota consist of the microorganisms that inhabit a host and include bacteria, fungi, viruses, and archaea. Sites such as the skin, respiratory tract, gastrointestinal tract, and vagina are colonized by these organisms, which play critical roles in health and disease. Systems approaches and –omics, in particular, have been utilized extensively to analyze the microbiota, resulting in the adoption of the term ‘microbiome’ to represent the collection of these organisms in a host. The microbiome affects everything from obesity, to resistance to colonization by pathogenic bacteria, to cancer (Ridaura et al., 2013; Petersen et al., 2019; Jacobson et al., 2018; Iida et al., 2013; Buffie et al., 2015; van Nood et al., 2013; Viaud et al., 2013; Sivan et al., 2015; Vetizou et al., 2015; Gopalakrishnan et al., 2018; Routy et al., 2018; Tanoue et al., 2019; Matson et al., 2018; Garrett, 2015; Reticker-Flynn and Engleman, 2019). While the microbiome mediates some of these effects directly (e.g. production of short-chain fatty acids), many are the indirect result of effects upon the immune system (Tanoue et al., 2019; Honda and Littman, 2016; Mazmanian et al., 2005; Atarashi et al., 2017; Atarashi et al., 2013; Ivanov et al., 2009; Gaboriau-Routhiau et al., 2009). In an effort to identify specific microbial components that are capable of influencing the adaptive immune response, researchers have colonized germ-free mice with feces from healthy human donors. Through 16S rRNA sequencing of the caecal contents of mice exhibiting enrichment of particular T cell subsets in their colons following fecal transplant or in comparison to mice under different housing conditions, they were able to define commensal consortia capable of inducing expansion of Tregs (Atarashi et al., 2013), T_H_1 (Atarashi et al., 2017), T_H_17 (Ivanov et al., 2009; Gaboriau-Routhiau et al., 2009), or IFN-γ^+^ CD8^+^ T cells (Tanoue et al., 2019). The last of these resulted in expansion of the T cells not only within the intestines, but also throughout the host in a manner that was protective against *Listeria monocytogenes* and augmented the efficacy of ICB. Similarly, among tumor-bearing mice, a comparison of germ-free to specific pathogen-free mice or mice from different vendors demonstrated differences in tumor growth depending on the microbiota. Moreover, specific commensals affected responses to ICB, with *Bifidobacterium* aiding anti-PD-1 (Sivan et al., 2015) and *Bacteroidales fragilis* aiding anti-CTLA-4 (Vetizou et al., 2015). To investigate whether the microbiome influences responses to ICB in humans, researchers characterized the diversity and composition of the microbiomes of patients receiving these therapies. These results showed that increased diversity and enrichment for Ruminococcaceae family bacteria correlate with improved response to anti-PD-1 and increases in CD8^+^ T cell activity (Gopalakrishnan et al., 2018) and that *Bifidobacterium longum*, *Collinsella aerofaciens*, and *Enterococcus faecium* may also augment responses by expanding tumor-specific CD8^+^ T cells without affecting Treg numbers (Matson et al., 2018). Similarly, fecal transplant into mice from human ICB responders resulted in increased efficacy of PD-1 blockade compared to that from non-responders, possibly due to the effects on recruitment of a CD4^+^ T cell subset (Routy et al., 2018). The ability to identify defined microbiota capable of improving responses to tumor immunotherapy is extremely exciting and would have been highly challenging without the –omics-level profiling technologies. It remains unclear, however, precisely how changes in the intestinal microbiota elicit systemic changes in adaptive immunity that extend beyond the gastrointestinal tract and against antigens that are not being presented there. Systems approaches will be critical to reveal the mechanisms by which this antigen-specific systemic immunity is established in response to alterations in the microbiota. Ultimately, these investigations are likely to lead to new ways of improving patient responses to ICB and possibly to altogether novel immunotherapies.

### Systems approaches to designing, monitoring, and evaluating clinical responses to tumor immunotherapy

**Figure 4.**
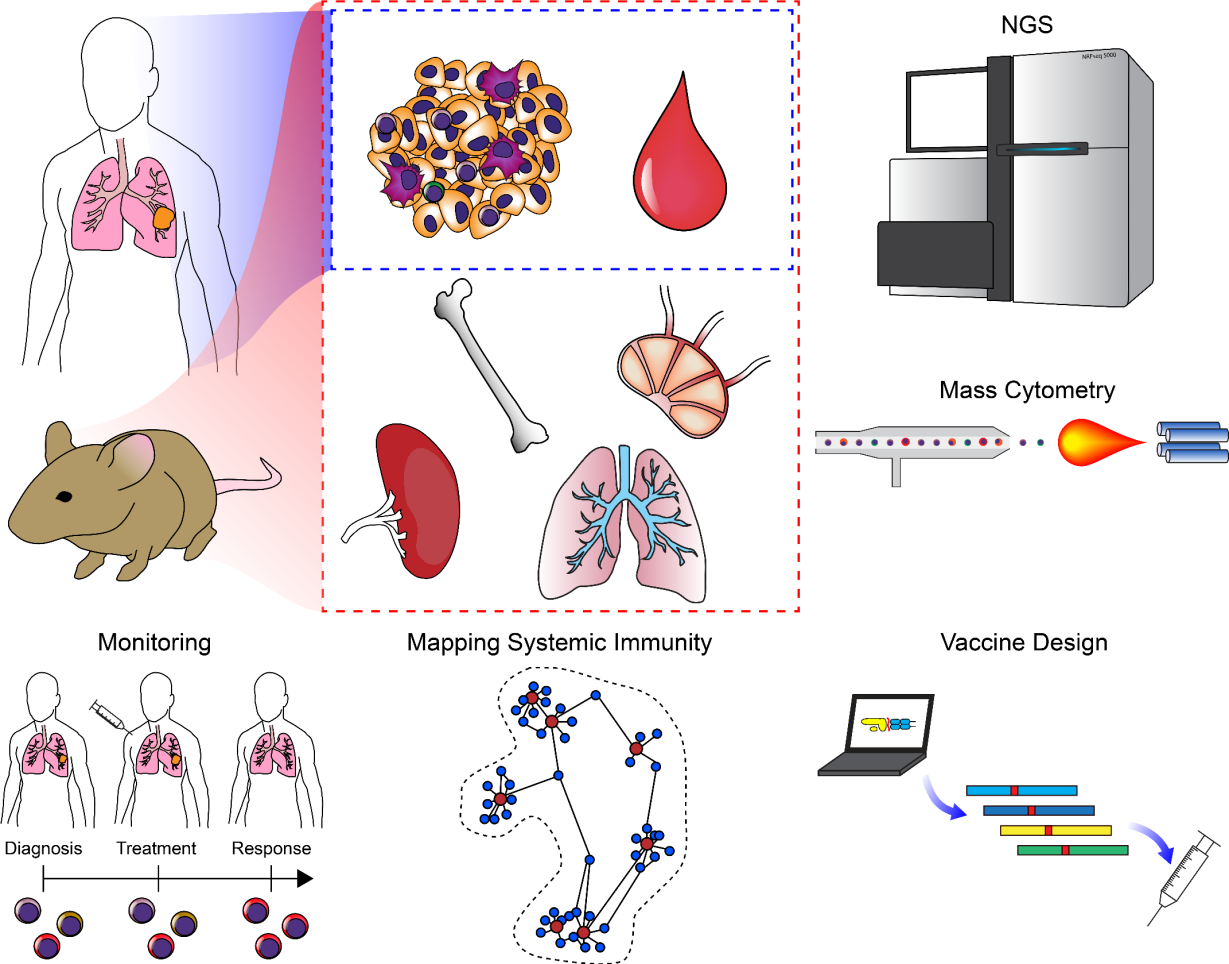
Clinical applications of cancer systems immunology. Tumor and peripheral blood samples are frequently harvested from patients in a longitudinal fashion. Model organisms such as mice enable researchers to query as many tissues as desired. These samples can be subjected to high-dimensional analysis platforms such as various NGS modalities or mass cytometry. Results from these analyses have been used to monitor responses to ICB (including immune repertoire analysis), mapping changes in the immune system across an organism, and for the design of personalized neoantigen vaccines.

Cancer immunotherapy, and ICB in particular, represents one of the most significant advances in oncology in decades and has the ability to elicit durable responses and cures in some patients with advanced stage malignancies (Rosenberg and Restifo, 2015; Brahmer et al., 2012; Hamid et al., 2013; Hodi et al., 2010; Nghiem et al., 2016; Pardoll, 2012; Robert et al., 2015; Sharma and Allison, 2015; Rizvi et al., 2015b; Grupp et al., 2013; Brentjens et al., 2013; Porter et al., 2011; June et al., 2018). Nonetheless, while subsets of patients with melanoma, RCC, Hodgkin’s lymphoma, NSCLC, urothelial cancer, and MSI high GI cancers have experienced durable responses and even complete tumor regression (Zappasodi et al., 2018), the majority of patients who receive the therapy do not exhibit such responses. Thus, there is an urgent need to distinguish in advance of treatment which patients will respond to which therapies as well as learn why certain patients exhibit durable responses while others either fail to respond at all or relapse after varying periods of response. The tools and approaches used by systems biologists to investigate tumor immune biology have enabled a variety of new approaches for designing and evaluating clinical responses. In many ways, most clinical practice could be construed as systems biology. Clinicians integrate cellular and molecular blood biomarkers (e.g. blood chemistry, complete blood counts, hematocrit, serum immunoglobulin levels, etc.), imaging, and biophysical measurements (e.g. temperature, blood pressure, etc.) in a longitudinal manner to gain an understanding of disease progression. Thus, the practice of medicine epitomizes the multiscale integrated analyses that lie at the core of systems biology. Indeed, pharmaceutical companies have used systems biology approaches in drug discovery and clinical trial design (Butcher et al., 2004). This holistic integration of multi-level datasets and the quantitation tools that accompany them render medicine amenable to the recent advances in cancer systems immunology and their application to evaluating tumor immunotherapy (Figure 4).

Many of the systems approaches discussed in this review are now being applied to advancing our understanding of the effects of existing immunotherapies and designing new approaches. As discussed in the previous section, researchers and clinicians have combined WES of patient tumors with HLA-binding and TCR prediction algorithms to design personalized neoantigen peptide vaccines (Figure 4; Ott et al., 2017; Hu et al., 2017b; Keskin et al., 2019). Staining for targeted costimulatory molecules (e.g. PD-L1) on tumors has exhibited variable predictive power in ICB, showing positive correlations in many studies but failing to predict responders whose tumors appear negative for the markers (Topalian et al., 2012; Nishino et al., 2017). In addition to the utility of WES in vaccine design, evaluation of neoantigen burden by WES has revealed that the response to anti-CTLA-4 (Snyder et al., 2014) and anti-PD1 (Rizvi et al., 2015a) therapies correlates with mutational burden. Recent studies have sought to exploit mass cytometry to develop high-dimensional cellular biomarkers of treatment response to ICB (Krieg et al., 2018; Wistuba-Hamprecht et al., 2017). One study identified not only differences in lymphocyte populations but also evidence of classical monocyte activation in PBMCs of patients who responded to anti-PD1 (Krieg et al., 2018). Additionally, standardized mass cytometry-based immunophenotyping approaches have been developed for monitoring clinical responses to immunotherapies (Hartmann et al., 2019), and this approach is currently being applied to monitoring responses to DC vaccination trials (Nowicki et al., 2019). Similarly, scRNA-seq approaches are being considered for monitoring immune responses to therapies and evaluating tumor heterogeneity, and will become increasingly feasible with the continual reduction in cost of these technologies (Shalek and Benson, 2017; Kim et al., 2016).

In addition to monitoring therapy in patients, systems approaches can reveal underlying biology about responses to treatment. A recent study revealing the clonal replacement of exhausted T cells following ICB is one such example demonstrating the power of integrative systems analyses to improve our understanding of the mechanisms of ICB (Figure 4; Yost et al., 2019). We believe that it is important to gain a holistic understanding of how immunity is orchestrated across an organism in order to understand the basis of immune responses in cancer. By combining our visualization method known as Statistical Scaffold with mass cytometry, we were able to generate an organism-wide map of differences in immune responses that distinguish effective from ineffective immunotherapy (Spitzer et al., 2017). This approach allowed us to compare immune repertoires in distinct tissues to each other and across treatment conditions, facilitating a systems-level understanding of immune responses across an organism. In addition to identifying differences in key immune populations such as a subset activated memory CD4^+^ T cells, we found that the key differentiator of effective immune responses is its orchestration from extratumoral sites such as LNs rather than a simple reinvigoration of existing TILs. Information gleaned from these types of systems-level analyses should facilitate the design of approaches that augment our current arsenal of immunotherapies (Figure 4).

### Expanding the future potential of cancer systems immunology

Technological and computational advances in the life sciences and the profound clinical successes of tumor immunotherapy have ushered the fields of systems biology and tumor immunology to the forefront of biomedical research. Understandably, the application of systems biology to tumor immunology is intuitive and has generated considerable excitement. Once lauded as representing the future of optimal cancer treatment and potential cures, personalized medicine fell out of fashion with the failure of targeted therapies to realize their promise (Nature Biotechnology, 2012). While –omics technologies bolstered the original enthusiasm for the field, they were ultimately applied in a reductionist fashion. Personalized medicine in cancer used to be construed as identifying oncogenic mutations and designing and treating with targeted small molecule or biological inhibitors of these mutations. These approaches demonstrated the innate shortcoming of reductionist analyses; that is, they fail to account for the inherent complexity of the tumor and its context within the patient. Escape mechanisms can involve a myriad of molecules and cell types requiring systems-level analyses to understand the emergent behavior of the tumor and its interactions with other cell types. In contrast to targeted therapies, immunotherapy exhibits the capacity, in many instances, to evolve with the tumor. Furthermore, it is capable of addressing the multifaceted components of the tumor that confer its malignant potential. The inherent systems-level nature of antitumor immunity thus requires a complementary set of approaches to interrogate the responsible immune cells and molecules, and to inform the design and implementation of new and existing therapies. Indeed, personalized medicine is experiencing a resurgence due to the potential of immunotherapies such as CAR-T cells and neoantigen vaccines to enable tumor targeting with a greater precision than previously possible. With an increased understanding of the dynamics underlying the coevolution of tumors and their immune responses during additional immunotherapies, such as ICB, systems biology will enhance our capacity to design and deliver personalized care to patients with advanced malignancies.

Cancer systems immunology is at an important transition point in its maturation. To date, preclinical and clinical studies exploiting systems approaches have generated profound amounts of data enabling characterization of immune responses and the generation of data rich atlases and accompanying tools for analysis. These important advances have laid the groundwork for discovery in the field of tumor immunology. The challenge moving forward will be to expand these approaches to uncover new biology that can be functionally validated. Many of the studies in the field have been largely descriptive and devoid of functional validation. In order to ensure that the field does not fall short of its promise, cancer systems immunologists will need to meet the higher bar of not only characterizing differences in immune responses to tumors but also performing the requisite experiments to determine the significance of the findings. Compared to other fields in cancer, this task is considerably more challenging. By definition, systems immunology typically uncovers interactions that involve many cellular components operating across multiple organ systems and timescales making perturbation of the components in a physiologically relevant manner difficult. Investigations in immunocompetent animal models, despite their challenges, represent some of the best approaches for testing the hypotheses generated from systems analyses. These models have served as the backbone for many discoveries in the field of immunology and will be necessary for advancing our understanding of tumor immunology. Future studies in the field should utilize advances in modeling approaches, such as ABM, to inform preclinical studies and focus the parameter space to one that is experimentally feasible. In some instances, where animal models are incapable of predicting patient responses, the use of systems approaches to integrate data sets and model human biology can inform the design of new therapies. Despite the challenges that confront the field, cancer systems immunology will continue to provide discoveries that lead to the next generation of life-saving immunotherapies.
